# Supplementary material for: DNA binding and bridging by human CtIP in the healthy and diseased states
Source: Nucleic Acids Res. 2024 Jun 26;52(14):8303–19. doi: 10.1093/nar/gkae538 (PMC11317151; doi:10.1093/nar/gkae538)
Supplement: gkae538_Supplemental_File [file gkae538_supplemental_file.pdf]

Supplementary Information

DNA binding and bridging by human CtIP in the healthy and diseased states

Shreya Lokanathan Balaji[1†], Sara De Bragança[2†], Francisco Balaguer-Pérez[2†], Sarah Northall[1†], Oliver Wilkinson[1], Clara Aicart-Ramos[2], Neeleema Seetaloo[3], Frank Sobott[3], Fernando Moreno-Herrero[2*] and Mark Simon Dillingham[1*]

[1] DNA:Protein Interactions Unit, School of Biochemistry, University of Bristol, BS8 1TD, UK.

[2] Department of Macromolecular Structures, Centro Nacional de Biotecnología, Consejo Superior de Investigaciones Científicas, 28049, Spain.

[3] Astbury Centre for Structural Molecular Biology and School of Molecular and Cellular Biology, University of Leeds, Leeds LS2 9JT, UK.

* To whom correspondence should be addressed. Email: fernando.moreno@cnb.csic.es and mark.dillingham@bristol.ac.uk

† Joint Authors

Supplementary Information

Including links/DOI for data access, Supplementary Methods, Supplementary Tables 1 and 2, Supplementary Figures 1-8 and Supplementary References.

Links/DOI for data access

The data underlying this article are available at Zenodo using digital object identifiers as follows:

Figures 2-3: DOI 10.5281/zenodo.10497388

https://zenodo.org/records/10497389

Figures 4 to 7 and Supplementary Figures S3 to S8: DOI 10.5281/zenodo.11371887

https://zenodo.org/records/11371887

Supplementary Methods

Preparation of DNA substrates for C-trap experiments

C-Trap experiments were performed on two types of molecules: a dsDNA molecule of 25427 bp and a loop-containing dsDNA consisting on a dsDNA tether of 21915 bp with a central loop of 18968 bp.

The linear dsDNA molecule was prepared as follows. The central part was obtained by digestion of a large homemade plasmid with BssHII (New England Biolabs) produced following published protocols (1). Without further purification, the fragment was ligated to highly biotinylated handles of ~1 kbp ending in MluI. Handles for C-trap constructs were prepared by PCR (see **Supplementary Table 1** for primers) including 200 μM final concentration of each dNTP (dGTP, dCTP, dATP), 140 μM dTTP and 66 μM Bio-16-dUTP (Roche) using the plasmid pSP73-JY0 as template (2) followed by digestion with the restriction enzyme MluI (New England Biolabs). Labelled handles were ligated with the central part with T4 DNA Ligase (New England Biolabs) for 15 h at 16°C followed by 1 h at 37°C before heat inactivation, in the presence of BssHII enzyme to avoid tandem (double-length) tethers. These handles were highly biotinylated to facilitate the capture of DNA molecules in C-Trap experiments. The sample was ready for use without further purification. DNAs were never exposed to intercalating dyes or UV radiation during their production and were stored at 4˚C with 1 mM EDTA pH 8.0 to final concentration to preserve them (see **Supplementary Table 2** for sequence).

The loop-containing DNA was prepared as follows. Two equal long dsDNA fragments of 7728 bp labelled with biotins in one end and containing two single-stranded regions in the opposite end, were annealed into the gaps created in a large homemade plasmid. This large plasmid is the same than the one employed above to fabricate the linear dsDNA substrate, and contains two regions of five spaced BbvCI restriction sites (see **Supplementary Table 2** for sequence). These poly-BbvCI regions were extracted from the pNLrep plasmid (kindly gifted by Prof. Dr. Ralf Seidel) and they are the same than the ones present in the plasmid employed to fabricate the magnetic tweezers DNA substrate, but separated by 6459 bp. The large plasmid was digested with the restriction enzyme Nt.BbvCI (New Englands Biolabs). This produced two sets of five nicks on one of the strands leading to two 63-nt gaps during heat inactivation of the nicking enzyme (3). This plasmid with two gaps was employed in a

later step without further purification. The 7728 bp-long dsDNA fragments were produced by PCR amplification with Phusion High-Fidelity DNA Polymerase (Thermo Scientific) using pSP73-JY0 as DNA template (2) (see **Supplementary Table 1** for primers) followed by purification (QIAGEN). The PCR fragment of 7688 bp contains a BbvCI restriction site in each end. Therefore, it was digested with Nt.BbvCI restriction enzyme creating a 5´-overhang of 13 nt in one end and a 3´-overhang of 12 nt in the opposite end. This simpler strategy to generate both 3´ and 5´-overhangs by using nicking enzymes has been previously described (3, 4). Without further purification, the 5´-overhang was filled in by DNA polymerase (Klenow Fragment [3′-5′ exo-], New England Biolabs) employing dGTP, dATP, biotin-16-dUTP and biotin-14-dCTP (Thermo Fisher Scientific) for 1 h at 37˚C. Reaction was followed by heat inactivation of the enzyme for 20 min at 75˚C. After that, the 3´-overhang of 12 nt was annealed with a small branched connector. This small branched connector was produced by annealing two partially complementary oligonucleotides (**Supplementary Table 1**) by heating at 95°C for 5 minutes and cooling down to 20°C at a 1°C $minute^{-1}$ rate in hybridization buffer (10 mM Tris-HCl pH 8.0, 1 mM EDTA, 200 mM NaCl, 5 mM $MgCl_2$). This small branched connector contains a 40-bp duplex stem ending in a 3´-overhang of 12 nt complementary to the 3´-overhang of the long dsDNA fragment, and two single-stranded regions complementary to the gaps created by Nt.BbvCI in the large plasmid. A 25X excess of the small branched connector was annealed into the 3´-overhang of the long dsDNA fragment by heating 10 min at 72°C, and slowly cooling down to 42°C at a 0.1°C every 25 sec rate in annealing buffer (10 mM Tris-HCl pH 7.5, 1 mM $MgCl_2$) followed by overnight with T4 DNA Ligase (New England Biolabs). The complete long dsDNA fragments already labelled with biotins in one end and with two single-stranded regions in the opposite end were gel extracted and purified (QIAGEN). Finally, a 5.4X excess of these long dsDNA fragments was hybridized into the gaps previously created in the large plasmid by heating 5 min at 80°C, and slowly cooling down to 30°C at a 0.5°C min^{-1} rate (but increasing 10 sec every minute) in annealing buffer (50 mM Tris-HCl pH 8.0, 1 mM EDTA, 100 mM NaCl) as described in (5). A final step of overnight ligation with T4 DNA Ligase was performed to seal all the nicks. The sample was ready for use without further purification. As usual, DNAs were never exposed to intercalating dyes or UV radiation during their production and were stored at 4˚C with EDTA pH 8.0 to 1 mM final concentration to preserve them.

For the binding of labelled short DNAs *in trans*, three types of molecules were prepared: two fork DNA molecules labelled with ATTO488 or Cy5, and a dsDNA small fragment of 139 bp labelled with ATTO488.

ATTO488-Fork and Cy5-Fork DNA molecules were prepared by annealing two partially complementary oligonucleotides (see **Supplementary Table 1**) by heating at 95°C for 5 minutes and cooling down to 20°C at a 1°C minute^{-1} rate in hybridization buffer (10 mM Tris-HCl pH 8.0, 1 mM EDTA, 200 mM NaCl, 5 mM $MgCl_2$). ATTO488-Fork molecule features two 37 nt-long polydTs with an ATTO488 label in the 5´-end of one of the branches, linked to a 26 bp-long dsDNA stem. Cy5-Fork molecule features two single-stranded regions of 31 and 32 nucleotides linked to a 60 bp-long dsDNA stem labelled with Cy5 in the 5´-end. Without further purification, samples were stored at -20°C.

139 bp-long ATTO488-dsDNA molecules randomly labelled with ATTO488 were prepared by PCR with GoTaq® G2 Flexi DNA polymerase (Promega) (see **Supplementary Table 1** for primers) including 200 µM final concentration of each dNTP (dGTP, dCTP, dATP), 180 µM dTTP and 20 µM Aminoallyl-dUTP-XX-ATTO488 (JenaBioscience) using the plasmid pSP73-JY0 as template (2). The PCR fragment was gel extracted, purified and stored at -20°C. DNAs were never exposed to intercalating dyes or UV radiation during their production.

Supplementary Table 1 – Sequences of oligonucleotides used in this study

Fragment	Oligonucleotide	Sequence
MT DIG handle	57.FMH_F2_BamHI-ApaI	GCGTAAGTGGATCCGGGCCCCGACTCACTATAGGGAGACCGGC
	JOE_R1	AGTAAGCGCCGTCAGACCAG
MT BIO handle	FMH_F2_BsrGI	GCGTAAGTTGTACACGACTCACTATAGGGAGACCGGC
	209.BsrGI 71short handle	CGATAACCAACTGGCGATG
C-Trap BIO handle	207.FMH_F2_MluI	GCGTAAGTACGCGTCGACTCACTATAGGGAGACCGGC
	JOE_R1	AGTAAGCGCCGTCAGACCAG
Forked-end branch	94.P-Fork 20T EcoRV NotI	(Pho)TCAGCTCATGTCATCCTCAGCACACTTGACCGGCTAGGCAGGATATCATGCATGCGGCCGCCATGGGAGGGTTTTTTTTTTTTTTTTTTT
	95.Fork 20T EcoRV NotI	TTTTTTTTTTTTTTTTTTTTCCCTCCCATGGCGGCCGCATGCATGATATCCTGCCTAGCCCTCAGCTCAGCTAGCCTCAGCCTACAATCACC
Cy5/HEX Fork DNA	Fork F	[Cy5]/[HEX]GCTTGCTAGGACGGATCGCTCGAGGTTTTTTTTTTTTTTTTTTT
	Fork R	TTTTTTTTTTTTTTTTTTTTCCTCGAGCGATCCGTCCTAGCAAGC
PCR of long dsDNA fragments	230.F ori BbvCI	GGCGGCGAGCTGAGGGTTACCGGATAAGGCGCAGCG
	231.R ori BbvCI	CCATGCATGCCCTCAGCCTGGCAGCAGCCACTGGTAAC
Small branched connector	232.P-12nt overhang EcoRV	[Pho]TCAGCTCATGTCATCCTCAGCACACTTGACCGGCTAGGCAGGATATCATGCATGCGGACGCCATGGGAGGGCCATGCATGCCC
	233.P-12nt overhang EcoRV	[Pho]CCCTCCCATGGCGTCCGCATGCATGATATCCTGCCTAGCCCTCAGCTCAGCTAGCCTCAGCCTACAATCACC
ATTO488-Fork	379.Fork with 243	GCTCATGTCATCCTCAGCACACTTGATTTTTTTTTTTTTTTTTTTTTTTTTTTTTTTTTTTT
	243.ATTO488 37Poly-T Nb.BbvCI	[ATTO488]TTTTTTTTTTTTTTTTTTTTTTTTTTTTTTTTTTTTTCAAGTGTGCTGAGGATGACATGAGC
Cy5-Fork	260.No P.96.Blunt-HindIII end	TCAGCTCATGTCATCCTCAGCACACTTGACCGGCTAGGCAGGATATCATGCATGCGGCCGCCATGGGAGGGTTAAGTAAGTAGATCAGAGA
	255.Cy5 blunt end	[Cy5]TCTCTGATCTACTTACTTAACCCTCCCATGGCGGCCGCATGCATGATATCCTGCCTAGCCCTCAGCTCAGCTAGCCTCAGCCTACAATCACC

ATTO488-dsDNA	FMH_F2	CGACTCACTATAGGGAGACCGGC
	209.BsrGI 71short handle	CGATAACCAACTGGCGATG

Supplementary Table 2. dsDNA constructs used in this study

- Underlined: 63 nt-gaps created after digestion with the nicking enzyme Nt.BbvCI followed by denaturation.
- Blue: 5´-overhang of 13 nt that is filled in by DNA polymerase (Klenow Fragment [3′-5′ exo-]) with biotinylated dNTPs.
- Orange: 3´-overhang of 12 nt that is annealed with the small branched connector.

**Fragment**	**Sequence**
MT central fragment<br>(4292 bp)	GTACAGAAAACTCACGTTAAGGGATTTTGGTCATGAGATTATCAAAAAGGATCTTCACCTAGATCCTTTTAAAT<br>TAAAAATGAAGTTTTAAATCAATCTAAAGTATATATGAGTAAACTTGGTCTGACAGTTACCAATGCTTAATCAG<br>TGAGGCACCTATCTCAGCGATCTGTCTATTTCGTTCATCCATAGTTGCCTGACTCCCCGTCGTGTAGATAACTA<br>CGATACGGGAGGGCTTACCATCTGGCCCCAGTGCTGCAATGATACCGCGAGACCCACGCTCACCGGCTCCAGAT<br>TTATCAGCAATAAACCAGCCAGCCGGAAGGGCCGAGCGCAGAAGTGGTCCTGCAACTTTATCCGCCTCCATCCA<br>GTCTATTAATTGTTGCCGGGAAGCTAGAGTAAGTAGTTCGCCAGTTAATAGTTTGCGCAACGTTGTTGCCATTG<br>CTACAGGCATCGTGGTGTCACGCTCGTCGTTTGGTATGGCTTCATTCAGCTCCGGTTCCCAACGATCAAGGCGA<br>GTTACATGATCCCCCATGTTGTGCAAAAAAGCGGTTAGCTCCTTCGGTCCTCCGATCGTTGTCAGAAGTAAGTT<br>GGCCGCAGTGTTATCACTCATGGTTATGGCAGCACTGCATAATTCTCTTACTGTCATGCCATCCGTAAGATGCT<br>TTTCTGTGACTGGTGAGTACTCAACCAAGTCATTCTGAGAATAGTGTATGCGGCGACCGAGTTGCTCTTGCCCG<br>GCGTCAATACGGGATAATACCGCGCCACATAGCAGAACTTTAAAAGTGCTCATCATTGGAAAACGTTCTTCGGG<br>GCGAAAACTCTCAAGGATCTTACCGCTGTTGAGATCCAGTTCGATGTAACCCACTCGTGCACCCAACTGATCTT<br>CAGCATCTTTTACTTTCACCAGCGTTTCTGGGTGAGCAAAAACAGGAAGGCAAAATGCCGCAAAAAAGGGAATA<br>AGGGCGACACGGAAATGTTGAATACTCATACTCTTCCTTTTTCAATATTATTGAAGCATTTATCAGGGTTATTG<br>TCTCATGAGCGGATACATATTTGAATGTATTTAGAAAAATAAACAAATAGGGGTTCCGCGCACATTTCCCCGAA<br>AAGTGCCACCTAAATTGTAAGCGTTAATATTTTGTTAAAATTCGCGTTAAATTTTTGTTAAATCAGCTCATTTT<br>TTAACCAATAGGCCGAAATCGGCAAAATCCCTTATAAATCAAAAGAATAGACCGAGATAGGGTTGAGTGTTGTT<br>CCAGTTTGGAACAAGAGTCCACTATTAAAGAACGTGGACTCCAACGTCAAAGGGCGAAAAACCGTCTATCAGGG<br>CGATGGCCCACTACGTGAACCATCACCCTAATCAAGTTTTTTGGGGTCGAGGTGCCGTAAAGCACTAAATCGGA<br>ACCCTAAAGGGAGCCCCCGATTTAGAGCTTGACGGGGAAAGCCGGCGAACGTGGCGAGAAAGGAAGGGAAGAAA<br>GCGAAAGGAGCGGGCGCTAGGGCGCTGGCAAGTGTAGCGGTCACGCTGCGCGTAACCACCACACCCGCCGCGCT<br>TAATGCGCCGCTACAGGGCGCGTCCCATTCGCCATTCAGGCTGCGCAACTGTTGGGAAGGGCGATCGGTGCGGG<br>CCTCTTCGCTATTACGCCAGCTGGCGAAAGGGGGATGTGCTGCAAGGCGATTAAGTTGGGTAACGCCAGGGTTT<br>TCCCAGTCACGACGTTGTAAAACGACGGCCAGTGAGCGCGCGTAATACGACTCACTATAGGGCGAATTGGGTAC<br>CGCTTGATAACGATTTGCTGAACACACCAGTGTAAGGGATGTTTATGACGAGCAAAGAAACCTTTACCCATTAC<br>CAGCCGCAGGGCAACAGTGACCCGGCTCATACCGCAACCGCGCCCGGCGGATTGAGTGCGAAAGCGCCTGCAAT<br>GACCCCGCTGATGCTGGACACCTCCAGCCGTAAGCTGGTTGCGTGGGATGGCACCACCGACGGTGCTGCCGTTG<br>GCATTCTTGCGGTTGCTGCTCGAGCCTCAGCTCATGTCATCCTCAGCACACTTGACCCTCAGCTCAGCTAGCCT<br>CAGCCTACAATCACCTCAGCGAATTCGGTGACCCTTACGCGAATCCGCTTTCAGACGTTGACTGGTCGCGTCTG<br>GCAAAAGTTAAAGACCTGACGCCCGGCGAACTGACCGCTGAGTCCTATGACGACAGCTATCTCGATGATGAAGA<br>TGCAGACTGGACTGCGACCGGGCAGGGGCAGAAATCTGCCGGAGATACCAGCTTCACGCTGGCGTGGATGCCCG<br>GAGAGCAGGGGCAGCAGGCGCTGCTGGCGTGGTTTAATGAAGGCGATACCCGTGCCTATAAAATCCGCTTCCCG<br>AACGGCACGGTCGATGTGTTCCGTGGCTGGGTCAGCAGTATCGGTAAGGCGGTGACGGCGAAGGAAGTGATCAC<br>CCGCACGGTGAAAGTCACCAATGTGGGACGTAACGATTTGCTGAACACACCAGTGTAAGGGATGTTTATGACGA<br>GCAAAGAAACCTTTACCCATTACCAGCCGCAGGGCAACAGTGACCCGGCTCATACCGCAACCGCGCCCGGCGGA<br>TTGAGTGCGAAAGCGCCTGCAATGACCCCGCTGATGCTGGACACCTCCAGCCGTAAGCTGGTTGCGTGGGATGG<br>CACCACCGACGGTGCTGCCGTTGGCATTCTTGCGGTTGCTGCTCGAGCCTCAGCTCATGTCATCCTCAGCACAC<br>TTGACCCTCAGCTCAGCTAGCCTCAGCCTACAATCACCTCAGCGAATTCGGTGACCCTTACGCGAATCCGCTTT<br>CAGACGTTGACTGGTCGCGTCTGGCAAAAGTTAAAGACCTGACGCCCGGCGAACTGACCGCTGAGTCCTATGAC<br>GACAGCTATCTCGATGATGAAGATGCAGACTGGACTGCGACCGGGCAGGGGCAGAAATCTGCCGGAGATACCAG<br>CTTCACGCTGGCGTGGATGCCCGGAGAGCAGGGGCAGCAGGCGCTGCTGGCGTGGTTTAATGAAGGCGATACCC<br>GTGCCTATAAAATCCGCTTCCCGAACGGCACGGTCGATGTGTTCCGTGGCTGGGTCAGCAGTATCGGTAAGGCG<br>GTGACGGCGAAGGAAGTGATCACCCGCACGGTGAAAGTCACCAATGTGGGACGTATCGAATTCCTGCAGCCCGG<br>GGATCCACTAGTTCTAGAGCGGCCGCCACCGCGGTGGAGCTCCAGCTTTTGTTCCCTTTAGTGAGGGTTAATT<br>GCGCGCTTGGCGTAATCATGGTCATAGCTGTTTCCTGTGTGAAATTGTTATCCGCTCACAATTCCACACAACAT

	ACGAGCCGGAAGCATAAAGTGTAAAGCCTGGGGTGCCTAATGAGTGAGCTAACTCACATTAATTGCGTTGCGCT<br>CACTGCCCGCTTTCCAGTCGGGAAACCTGTCGTGCCAGCTGCATTAATGAATCGGCCAACGCGCGGGGAGAGGC<br>GGTTTGCGTATTGGGCGCTCTTCCGCTTCCTCGCTCACTGACTCGCTGCGCTCGGTCGTTCGGCTGCGGCGAGC<br>GGTATCAGCTCACTCAAAGGCGGTAATACGGTTATCCACAGAATCAGGGGATAACGCAGGAAAGAACATGTGAG<br>CAAAAGGCCAGCAAAAGGCCAGGAACCGTAAAAAGGCCGCGTTGCTGGCGTTTTTCCATAGGCTCCGCCCCCCT<br>GACGAGCATCACAAAAATCGACGCTCAAGTCAGAGGTGGCGAAACCCGACAGGACTATAAAGATACCAGGCGTT<br>TCCCCCTGGAAGCTCCCTCGTGCGCTCTCCTGTTCCGACCCTGCCGCTTACCGGATACCTGTCCGCCTTTCTCC<br>CTTCGGGAAGCGTGGCGCTTTCTCATAGCTCACGCTGTAGGTATCTCAGTTCGGTGTAGGTCGTTCGCTCCAAG<br>CTGGGCTGTGTGCACGAACCCCCCGTTCAGCCCGACCGCTGCGCCTTATCCGGTAACTATCGTCTTGAGTCCAA<br>CCCGGTAAGACACGACTTATCGCCACTGGCAGCAGCCACTGGTAACAGGATTAGCAGAGCGAGGTATGTAGGCG<br>GTGCTACAGAGTTCTTGAAGTGGTGGCCTAACTACGGCTACACTAGAAGGACAGTATTTGGTATCTGCGCTCTG<br>CTGAAGCCAGTTACCTTCGGAAAAAGAGTTGGTAGCTCTTGATCCGGCAAACAAACCACCGCTGGTAGCGGTGG<br>TTTTTTTGTTTGCAAGCAGCAGATTACGCGCAGAAAAAAAGGATCTCAAGAAGATCCTTTGATCTTTTCTACGG
C-trap central fragment of the linear dsDNA molecule. C-trap circular piece of the loop-containing DNA.<br><br>(25427 bp)	CGCGCATTGCGCCCAGCGCCATCTGATCGTTGGCAACCAGCATCGCAGTGGGAACGATGCCCTCATTCAGCATT<br>TGCATGGTTTGTTGAAAACCGGACATGGCACTCCAGTCGCCTTCCCGTTCCGCTATCGGCTGAATTTGATTGCG<br>AGTGAGATATTTATGCCAGCCAGCCAGACGCAGACGCGCCGAGACAGAACTTAATGGGCCCGCTAACAGCGCGA<br>TTTGCTGGTGACCCAATGCGACCAGATGCTCCACGCCCAGTCGCGTACCGTCTTCATGGGAGAAAATAATACTG<br>TTGATGGGTGTCTGGTCAGAGACATCAAGAAATAACGCCGGAACATTAGTGCAGGCAGCTTCCACAGCAATGGC<br>ATCCTGGTCATCCAGCGGATAGTTAATGATCAGCCCACTGACGCGTTGCGCGAGAAGATTGTGCACCGCCGCTT<br>TACAGGCTTCGACGCCGCTTCGTTCTACCATCGACACCACCACGCTGGCACCCAGTTGATCGGCGCGAGATTTA<br>ATCGCCGCGACAATTTGCGACGGCGCGTGCAGGGCCAGACTGGAGGTGGCAACGCCAATCAGCAACGACTGTTT<br>GCCCGCCAGTTGTTGTGCCACGCGGTTGGGAATGTAATTCAGCTCCGCCATCGCCGCTTCCACTTTTTCCCGCG<br>TTTTCGCAGAAACGTGGCTGGCCTGGTTCACCACGCGGGAAACGGTCTGATAAGAGACACCGGCATACTCTGCG<br>ACATCGTATAACGTTACTGGTTTCACATTCACCACCCTGAATTGACTCTCTTCCGGGCGCTATCATGCCATACC<br>GCGAAAGGTTTTGCGCCATTCGATGGTGTCCGGGATCTCGACGCTCTCCCTTATGCGACTCCTGCATTAGGAAG<br>CAGCCCAGTAGTAGGTTGAGGCCGTTGAGCACCGCCGCCGCAAGGAATGGTGCATGCAAGGAGATGGCGCCCAA<br>CAGTCCCCCGGCCACGGGGCCTGCCACCATACCCACGCCGAAACAAGCGCTCATGAGCCCGAAGTGGCGAGCCC<br>GATCTTCCCCATCGGTGATGTCGGCGATATAGGCGCCAGCAACCGCACCTGTGGCGCCGGTGATGCCGGCCACG<br>ATGCGTCCGGCGTAGAGGATCGAGATCTCGATCCCGCGAAATTAATACGACTCACTATAGGGGAATTGTGAGCG<br>GATAACAATTCCCTTCTAGCCATATTGGACTCGGACCTGTTTCACGTGGAACACTGAGCCTGGACTAGGTCTAG<br>CATATTGGACTCGACTTTCACGTGGAACACTGAGCCTGGACTAGGTCTAGAACAGGTTCTTTTTTCTTTGTTTC<br>ACGTGGAACATTCTGATTAATGTACAGCTAGCCATATTGGACTCGGACCTGTTTCACGTGGAACACTGAGCCTG<br>GACTAGGTCTAGAACAGGTTCTTTTTTCTTTGTTTCACGTGGAACATTCTGATTAATGTACAGCTAGAACAGGT<br>TCTTTTTTCTTTGTTTCACGTGGAACATTCTGATTAATGTACAGCTAGAAATAATTTTGTTTAACTTTAAGAAG<br>GAGATATACCATGTGTTTCACGTGGAACACATGGCTAAAGGCCTTGGAAAAGGGATTAATGCGTTATTTAATCA<br>GGTAGATTTGTCTGAAGAGACAGTTGAAGAAATTAAAATTGCCGATTTACGCCCTAATCCTTATCAGCCAAGAA<br>AACACTTTGATGACGAGGCATTAGCTGAACTAAAAGAATCTGTGCTGCAGCATGGCATTCTTCAGCCGCTTATC<br>GTCAGAAAATCTTTAAAAGGCTATGATATTGTTGCGGGTGAACGGCGTTTTCGAGCGGCAAAGCTGGCAGGTTT<br>AGATACAGTTCCGGCCATTGTCCGTGAATTATCAGAGGCGTTAATGAGGGAAATTGCTTTATTAGAAAACCTTC<br>AGCGTGAAGATTTATCTCCGCTTGAAGAGGCTCAGGCATATGCCGAAACAAGCGCTCATGAGCCCGAAGTGGCG<br>AGCCCGATCTTCCCCATCGGTGATGTCGGCGATATAGGCGCCAGCAACCGCACCTGTGGCGCCGGTGATGCCGG<br>CCACGATGCGTCCGGCGTAGAGGATCGAGATCTCGATCCCGCGAAATTAATACGACTCACTATAGGGGAATTGT<br>GAGCGGATAACAATTCCCTTCTAGCCATATTGGACTCGGACCTGTTTCACGTGGAACACTGAGCCTGGACTAGG<br>TCTAGCATATTGGACTCGACTTTCACGTGGAACACTGAGCCTGGACTAGGTCTAGAACAGGTTCTTTTTTCTTT<br>GTTTCACGTGGAACATTCTGATTAATGTACAGCTAGCCATATTGGACTCGGACCTGTTTCACGTGGAACACTGA<br>GCCTGGACTAGGTCTAGAACAGGTTCTTTTTTCTTTGTTTCACGTGGAACATTCTGATTAATGTACAGCTAGAA<br>CAGGTTCTTTTTTCTTTGTTTCACGTGGAACATTCTGATTAATGTACAGCTAGAAATAATTTTGTTTAACTTTA<br>AGAAGGAGATATACCATGTGTTTCACGTGGAACACATGGCTAAAGGCCTTGGAAAAGGGATTAATGCGCATATG<br>ACTCCCTTTTGAAACACTTAGATCTCACACAAGAGCAGCTTGCCAAACGTCTTGGGAAAAGCAGACCGCATATT<br>GCGAATCATTTAAGACTGCTGACACTGCCAGAAAATATTCAACAGCTTATTGCCGAAGGCACGCTTTCTATGGG<br>ACATGGACGCACGCTTCTTGGCTTAAAAAACAAAAATAAGCTTGAACCGCTGGTACAAAAAGTGATTGCGGAGC<br>AGCTCAATGTTCGCCAACTTGAGCAGCTGATTCAGCAGTTGAATCAGAATGTTCCACGTGAAACAAAGAAAAAA<br>GAACCTGTGAAAGATGCGGTTCTAAAAGAACGGGAATCCTATCTCCAAAATTATTTTGGAACAACAGTTAATAT<br>TAAAAGACAGAAGAAAAAAGGCAAAATCGAAATTGAATTTTTCTCTAATGAAGACCTTGACCGGATTTTAGAGC<br>TTTTGTCTGAACGAGAATCATAAGGATCCGAATTCATCCGAGAATTCCGATAGGAATTCCACGTTGAATTCTAC<br>GACGAATTCTCAACTGTGAGGAGGCCACGGTACTGGAGTCGTTTCTGGAAGAGCACGGGGGCTGGAAATCCTTT<br>CTGTGGACGCCGCCTTATGAGTGGCGGCAGATAAAGGTGACCTGCGCAAAATGGTCGTCGCGGGTCAGTATGCT<br>GCGTGTTGAGTTCAGCGCAGAGTTTGAACAGGTGGTGAACTGATGCAGGATATCCGGCAGGAAACACTGAATGA

	ATGCACCCGTGCGGAGCAGTCGGCCAGCGTGGTGCTCTGGGAAATCGACCTGACAGAGGTCGGTGGAGAACGTT<br>ATTTTTTCTGTAATGAGCAGAACGAAAAAGGTGAGCCGGTCACCTGGCAGGGGCGACAGTATCAGCCGTATCCC<br>ATTCAGGGGAGCGGTTTTGAACTGAATGGCAAAGGCACCAGTACGCGCCCCACGCTGACGGTTTCTAACCTGTA<br>CGGTATGGTCACCGGGATGGCGGAAGATATGCAGAGTCTGGTCGGCGGAACGGTGGTCCGGCGTAAGGTTTACG<br>CCCGTTTTCTGGATGCGGTGAACTTCGTCAACGGAAACAGTTACGCCGATCCGGAGCAGGAGGTGATCAGCCGC<br>TGGCGCATTGAGCAGTGCAGCGAACTGAGCGCGGTGAGTGCCTCCTTTGTACTGTCCACGCCGACGGAAACGGA<br>TGGCGCTGTTTTTCCGGGACGTATCATGCTGGCCAACACCTGCACCTGGACCTATCGCGGTGACGAGTGCGGTT<br>ATAGCGGTCCGGCTGTCGCGGATGAATATGACCAGCCAACGTCCGATATCACGAAGGATAAATGCAGCAAATGC<br>CTGAGCGGTTGTAAGTTCCGCAATAACGTCGGCAACTTTGGCGGCTTCCTTTCCATTAACAAACTTTCGCAGTA<br>AATCCCATGACACAGACAGAATCAGCGATTCTGGCGCACGCCCGGCGATGTGCGCCAGCGGAGTCGTGCGGCTT<br>CGTGGTAAGCACGCCGGAGGGGGAAAGATATTTCCCCTGCGTGAATATCTCCGGTGAGCCGGAGGCTATTTCCG<br>TATGTCGCCGGAAGACTGGCTGCAGGCAGAAATGCAGGGTGAGATTGTGGCGCTGGTCCACAGCCACCCCGGTG<br>GTCTGCCCTGGCTGAGTGAGGCCGACCGGCGGCTGCAGGTGCAGAGTGATTTGCCGTGGTGGCTGGTCTGCCGG<br>GGGACGATTCATAAGTTCCGCTGTGTGCCGCATCTCACCGGGCGGCGCTTTGAGCACGGTGTGACGGACTGTTA<br>CACACTGTTCCGGGCGGCCCCGCAAGGATTGCCCCGATGCCTTGTTCCTTTGCCGCGAGAATGGCGGCCAACAG<br>GTCATGTTTTTCTGGCATCTTCATGTCTTACCCCCAATAAGGGGATTTGCTCTATTTAATTAGGAATAAGGTCG<br>ATTACTGATAGAACAAATCCAGGCTACTGTGTTTAGTAATCAGATTTGTTCGTGACCGATATGCACGGGCAAAA<br>CGGCAGGAGGTTGTTAGCGCGACCTCCTGCCACCCGCTTTCACGAAGGTCATGTGTAAAAGGCCGCAGCGTAAC<br>TATTACTAATGAATTCAGGACAGACAGTGGCTACGGCTCAGTTTGGGTTGTGCTGTTGCTGGGCGGCGATGACG<br>CCTGTACGCATTTGGTGATCCGGTTCTGCTTCCGGTATTCGCTTAATTCAGCACAACGGAAAGAGCACTGGCTA<br>ACCAGGCTCGCCGACTCTTCACGATTATCGACTCAATGCTCTTACCTGTTGTGCAGATATAAAAAATCCCGAAA<br>CCGTTATGCAGGCTCTAACTATTACCTGCGAACTGTTTCGGGATTGCATTTTGCAGACCTCTCTGCCTGCGATG<br>GTTGGAGTTCCAGACGATACGTCGAAGTGACCAACTAGGCGGAATCGGTAGTAAGCGCCGCCTCTTTTCATCTC<br>ACTACCACAACGAGCGAATTAACCCATCGTTGAGTCAAATTTACCCAATTTTATTCAATAAGTCAATATCATGC<br>CGTTAATATGTTGCCATCCGTGGCAATCATGCTGCTAACGTGTGACCGCATTCAAAATGTTGTCTGCGATTGAC<br>TCTTCTTTGTGGCATTGCACCACCAGAGCGTCATACAGCGGCTTAACAGTGCGTGACCAGGTGGGTTGGGTAAG<br>GTTTGGGATTAGCATCGTCACAGCGCGATATGCTGCGCTTGCTGGCATCCTTGAATAGCCGACGCCTTTGCATC<br>TTCCGCACTCTTTCTCGACAACTCTCCCCCACAGCTCTGTTTTGGCAATATCAACCGCACGGCCTGTACCATGG<br>CAATCTCTGCATCTTGCCCCCGGCGTCGCGGCACTACGGCAATAATCCGCATAAGCGAATGTTGCGAGCACTTG<br>CAGTACCTTTGCCTTAGTATTTCCTTCAAGCTTTGCCACACCACGGTATTTCCCCGATACCTTGTGTGCAAATT<br>GCATCAGATAGTTGATAGCCTTTTGTTTGTCGTTCTGGCTGAGTTCGTGCTTACCGCAGAATGCAGCCATACCG<br>AATCCGGCTTGTGATTGCGCCATCCCCATAGCAGCCATCACATCAGTACCGGAAAGAGAGTCAGAAGCCGTGGC<br>CCGTGGTGAGTCGCTCATCATCGGGCTTTTTGGCGAATGAAATTTAGCTACGCTTTCGAGTCTCATGCGCCTTC<br>TCCCTGTACCTGAATCAATGTTAGGTTTCCGCAGAACACTGCGCCGGTATCGATATACATTTGGTTGGCAAACT<br>TGAGTGGTTTCACTGCTGGCGTATGACCAAAGATGAACGTGTCCGCGCCTTTGATTTCTTTCACGATCCCGTTT<br>TGTGAGTTGCTGATTCGTTCGCGGTTCCAGATTACCTGCTGATGATCAACTGGCTTTCCAAACTCGTATTCGTC<br>AAAGGGATAATCGGCGTGGCAGATAACATATTTTTTATCTTTGCTCACCAGTTCGATGATTAACGGAAGTTCAT<br>CTGCTTTATGGGCAAGAGCTTTAGCCAGAATTTCTTTGTCGTAATCGAGATTAAAGAACCAGCCACCGCCATTA<br>AGCAGCCAGTGATTAACGTTTCCACGCTCTGATAAGCCATCAATCATCATTTGCTCATGGTTTCCACGTACAGC<br>TCTGAACCAGGGGAATGTGATTAATTCCAGGCATTCAACGTTCTCTGCACCACGATCAACCAAATCGCCCACCG<br>AGATAAGCAGGTCTTTTTTGTTGTCGAATCCAATCGTATCCAGTTTGTTCATCAGGTTCGTGTAGCATCCGTGC<br>AGATCGCCAACTACCCAAATATTTCGGTATTTGCTGCCATCAATTTTTTCGTAATAGCGCATCTCTTTCACTCC<br>ATCCGCGATGAACCATGAGAACGTCGTTGACGATGGCGTGCATTTTCCCGTCTTTATCATCAACGTATTTTCTG<br>ACCGTACCGCGACTACATTTCAGTCTGCGTGCTACTTCTGTCTGATTTCCGTATGTTTCAACGAGCATGTCTGG<br>AATGGTTTTTACTGAGAACGTCATGCGGCCTCACTTCTGCTATTTCGCAGGTCTTTGAGTTTCTGTTGGTACTC<br>TGCCTTGATCGCCTTGCACTCTTCGATAGTCCAGCGATGGCGGTTATGGTTTGATTCGATTTCGTCTACTGCTT<br>CCTGCCCGATGCGGCTAATCAGTTCGACGCGATACGGAACGAGATTTCCGCTTTTGTGCTGGTTGCACACCACG<br>CATTGCTTGTGAATATTGCGTTCATTAAATCGGAGTTGAGGTGCCGCAGCAGTTGTCCGGTAATGTCCGGCATC<br>CCACTGAGCAGACGTGAGCGTTCCGCACGAGATACATGGTAAGTCGCGGTCTCTTTCTCTGATGAAGGCGTTTA<br>CGGCTTGTTGGGCTTGTTTAATCCAGTAACTGCGGGGCTTTAAGGCGAGTTTTCGAATCTTAAGTTTATCTTTC<br>TGTTTCTGCTCCTCTCGTCGTCGTTTCTTCTCTGCTGCTTTTTCCGCTTTTTCGCGTTCTTTACTTCGTCGTTC<br>GAGTGCTATCTTGGTTCCACACTCTGGAGAGCACCACCACTGATTAGCGAATGCAGGGTGAAACCATTCCCGGC<br>ATTCATCGTTTTTACATCGTCTTCGCGCTGGTTTAGCCATCATCTTCTTCCTCGTGCATCGAGCTATTCGGATC<br>GCTCATCAGTTCTGCGCAGCAGTGCTCACACACGTGAACTTCCAGCACATGCAGCTTCTGACCGCAGTTAGCGC<br>ACGTTAAAGCTCGCTCGACGCTTTCTTGTTCGTAACTTCGATTTTGGTCAATCACCTTGTTTTCCTCGCACGAC<br>GTCTTAGCCACCGGATATCCCACAGGTGAGCCGTGTAGTTGAAGGTTTTTACGTCAGATTCTTTTGGGATTGGC<br>TTGGGTTTATTTCTGGTGCGTTTCGTTGGAAGGTATTTGCAGTTTTCGCAGATTATGTCGGTGATACTTCGTCG<br>CTGTCTCGCCACACGTCCTCCTTTTCCTGCGGTAGTGGTAACACCCCTGTTGGTGTTCTTTCACACCGGAGACA<br>CCATCGATTCCAGTAAGGTTGATTTGGTCGGAAGCGGTTATCTTCTTTGCATTCACCGCACCGATAACATCGCA

	TCATGCAGCTTCCCTCCCGAAGTCGAAATCAAGCTGCCCTCCAAATATTTCGCATGACTCAGAACAAGAGCCGG<br>TATCGAATCTTTTAGCTCGTACCATGTCCTGATACAGGGCTTGATAATCATTTTCTGAATACATTTTCGCGATA<br>CCGTCCAGCGACATTCTTCCTCGGTACATAATCTCCTTTGGCGTTTCCCGATGTCCGTCACGCACATGGGATCC<br>CGTGATGACCTCATTAAAAACACGCTGCAATCCCTCCTCATCTTTGCAGGCAAGTCCGATTTTTTGCGTTGATT<br>TTTTAATGCAGAATATGCAGTTACCGAGATGTTCCGGTATTTGCAAATCGAATGGTTGTTGCTTCCACCATGCG<br>AGGATATCTTCCTTCTCAAAGTCTGACAGTTCAGCAAGATATCTGATTCCAGGCTTTGGCTTTAGCCGCTTCGG<br>TTCATCAGCTCTGATGCCAATCCACGTGGTGTAATTCCCTCGCCCGAAATGGTCATCACAGTATTTGGTGAAGG<br>GAACGAGTTTTAATCTGTCAGTGCAGAACGCGCCGCCGACGTATGGAGTGCCATATTTCTTTACCATATCGATA<br>AATGGCTTCAGAACAGGCATTCGCGTCTGAATATCCTTTGGTTCCCATACCGTATAACCATTTGGCTGTCCAAG<br>CTCCGGGTTGATATCAACCTGCAATACGGTGAGCGGTATATCCCAGAACTTCACAACTTCCCTGACAAACCGAT<br>ATGTCATTGGATGTTCACAACCTGTATCCATGAAAACGTAATGCACGTCTTTACCTGCCCGTCGCTTTTGCTCC<br>ATTAGCCAGAGCAAATATGCTGACGTCCTGCCACCGGAGAAACTAACGACATTTATCATGCAGCCCTGTCTCCC<br>CATCTCGCTTTCCACTCCAGAGCCAGTCTCGCTTCGTCTGACCACTTAACGCCACGCTCTGTACCGAATGCCTG<br>TATAAGCTCTAATAGCTCCGCAAATTCGCCTACACGCATCCTGCTGGTTGACTGGCCTATTACCACAAAGCCAT<br>TCCCGGCAAGGTTAGGAACAACATCCTGCTGCTTTAATGCTGCGGTAAACACACACTTCCAGCTTTCTGCATCC<br>AGCCAGCGACCATGCCATTCAACCTGACGAGAGACGTCACCTAAGCAGGCCCATAGCTTCCTGTTTTGGTCTAA<br>GCTGCGGTTGCGTTCCTGAATGGTTACTACGATTGGTTTGGTTGGGTCTGGAAGGATTTGCTGTACTGCGTGAA<br>TAGCGTTTTGCTGATGTGCTGGAGATCGAATTTCAAAGGTTAGTTTTTTCATGACTTCCCTCTCCCCCAAATAA<br>AAAGGCCTGCGATTACCAGCAGGCCTGTTATTAGCTCAGTAATGTAGATGGTCATCTTTTAACTCCATATACCG<br>CCAATACCCGTTTCATCGCGGCACTCTGGCGACACTCCTTAAAAACCAGGTTCGTGCTCATCTTTCCTTCCCGT<br>TCTTCCCTGGTAGCAAACCGGTAATACACCGTTCGCCAGACCTTACCTTCGATAACCAGAAGACCTGCCCGTGC<br>CATTTTAGCCGCGGCCTGATTTATGCTGGTTACTGTTGCGCCTGTTAGCGCGGCAACGTCCGGCGCACAGAAGC<br>TATTATGCGTCCCCAGGTAATGAATAATTGCCTCTTTGCCCGTCATACACTTGCTCCTTTCAGTCCGAACTTAG<br>CTTTGATTTCTGCGATCTTCGCCAGAGCCTGTGCACGATTTAGAGGTCTACCGCCCATGACAGGAAGTTGTTTT<br>ACTGGTTCAGGGATCGCCTCACCACGGTTAATTCTCGCAGTCATATGGACAAGCTCATCTGCGGCCTTACGGCG<br>TAATTCCGCATCAGTAAGCGCATTGGCCCGCATGTTCTGATACAGGTTGGTAACCAGCCAGTAGTGCGCGTTTG<br>ATTTCCACGGATAAGACTCCGCATCCGGCTCGAGCCTCAGCTCATGTCATCCTCAGCACACTTGACCCTCAGCT<br>CAGCTAGCCTCAGCCTACAATCACCTCAGCGAATTCGGTGACCCTTACGCGAATCCGCTTTCAGACGTTGACTG<br>GTCGCGTCTGGCAAAAGTTAAAGACCTGACGCCCGGCGAACTGACCGCTGAGTCCTATGACGACAGCTATCTCG<br>ATGATGAAGATGCAGACTGGACTGCGACCGGGCAGGGGCAGAAATCTGCCGGAGATACCAGCTTCACGCTGGCG<br>TGGATGCCCGGAGAGCAGGGGCAGCAGGCGCTGCTGGCGTGGTTTAATGAAGGCGATACCCGTGCCTATAAAAT<br>CCGCTTCCCGAACGGCACGGTCGATGTGTTCCGTGGCTGGGTCAGCAGTATCGGTAAGGCGGTGACGGCGAAGG<br>AAGTGATCACCCGCACGGTGAAAGTCACCAATGTGGGACGTCCGTCGATGGCAGAAGATCGCAGCACGGTAACA<br>GCGGCAACCGGCATGACCGTGACGCCTGCCAGCACCTCGGTGGTGAAAGGGCAGAGCACCACGCTGACCGTGGC<br>CTTCCAGCCGGAGGGCGTAACCGACAAGAGCTTTCGTGCGGTGTCTGCGGATAAAACAAAAGCCACCGTGTCGG<br>TCAGTGGTATGACCATCACCGTGAACGGCGTTGCTGCAGGCAAGGTCAACATTCCGGTTGTATCCGGTAATGGT<br>GAGTTTGCTGCGGTTGCAGAAATTACCGTCACCGCCAGTTAATCCGGAGAGTCAGCGATGTTCCTGAAAACCGA<br>ATCATTTGAACATAACGGTGTGACCGTCACGCTTTCTGAACTGTCAGCCCTGCAGCGCATTGAGCATCTCGCCC<br>TGATGAAACGGCAGGCAGAACAGGCGGAGTCAGACAGCAACCGGAAGTTTACTGTGGAAGACGCCATCAGAACC<br>GGCGCGTTTCTGGTGGCGATGTCCCTGTGGCATAACCATCCGCAGAAGACGCAGATGCCGTCCATGAATGAAGC<br>CGTTAAACAGATTGAGCAGGAAGTGCTTACCACCTGGCCCACGGAGGCAATTTCTCATGCTGAAAACGTGGTGT<br>ACCGGCTGTCTGGTATGTATGAGTTTGTGGTGAATAATGCCCCTGAACAGACAGAGGACGCCGGGCCCGCAGAG<br>CCTGTTTCTGCGGGAAAGTGTTCGACGGTGAGCTGAGTTTTGCCCTGAAACTGGCGCGTGAGATGGGGCGACCC<br>GACTGGCGTGCCATGCTTGCCGGGATGTCATCCACGGAGTATGCCGACTGGCACCGCTTTTACAGTACCCATTA<br>TTTTCATGATGTTCTGCTGGATATGCACTTTTCCGGGCTGACGTACACCGTGCTCAGCCTGTTTTTCAGCGATC<br>CGGATATGCATCCGCTGGATTTCAGTCTGCTGAACCGGCGCGAGGCTGACGTCGAGTGAAGCGTTATTGGTATG<br>CGGTAAAACCGCACTCAGGCGGCCTTGATAGTCATATCATCTGAATCAAATATTCCTGATGTATCGATATCGGT<br>AATTCTTATTCCTTCGCTACCATCCATTGGAGGCCATCCTTCCTGACCATTTCCATCATTCCAGTCGAACTCAC<br>ACACAACACCATATGCATTTAAGTCGCTTGAAATTGCTATAAGCAGAGCATGTTGCGCCAGCATGATTAATACA<br>GCATTTAATACAGAGCCGTGTTTATTGAGTCGGTATTCAGAGTCTGACCAGAAATTATTAATCTGGTGAAGTTT<br>TTCCTCTGTCATTACGTCATGGTCGATTTCAATTTCTATTGATGCTTTCCAGTCGTAATCAATGATGTATTTTT<br>TGATGTTTGACATCTGTTCATATCCTCACAGATAAAAAATCGCCCTCACACTGGAGGGCAAAGAAGATTTCCAA<br>TAATCAGAACAAGTCGGCTCCTGTTTAGTTACGAGCGACATTGCTCCGTGTATTCACTCGTTGGAATGAATACA<br>CAGTGCAGTGTTTATTCTGTTATTTATGCCAAAAATAAAGGCCACTATCAGGCAGCTTTGTTGTTCTGTTTACC<br>AAGTTCTCTGGCAATCATTGCCGTCGTTCGTATTGCCCATTTATCGACATATTTCCCATCTTCCATTACAGGAA<br>ACATTTCTTCAGGCTTAACCATGCATTCCGATTGCAGCTTGCATCCATTGCATCGCTTGAATTGTCCACACCAT<br>TGATTTTTATCAATAGTCGTAGTCATACGGATAGTCCTGGTATTGTTCCATCACATCCTGAGGATGCTCTTCGA<br>ACTCTTCAAATTCTTCTTCCATATATCACCTTAAATAGTGGATTGCGGTAGTAAAGATTGTGCCTGTCTTTTAA<br>CCACATCAGGCTCGGTGGTTCTCGTGTACCCCTACAGCGAGAAATCGGATAAACTATTACAACCCCTACAGTTT

	GATGAGTATAGAAATGGATCCACTCGTTATTCTCGGACGAGTGTTCAGTAATGAACCTCTGGAGAGAACCATGT<br>ATATGATCGTTATCTGGGTTGGACTTCTGCTTTTAAGCCCAGATAACTGGCCTGAATATGTTAATGAGAGAATC<br>GGTATTCCTCATGTGTGGCATGTTTTCGTCTTTGCTCTTGCATTTTCGCTAGCAATTAATGTGCATCGATTATC<br>AGCTATTGCCAGCGCCAGATATAAGCGATTTAAGCTAAGAAAACGCATTAAGATGCAAAACGATAAAGTGCGAT<br>CAGTAATTCAAAACCTTACAGAAGAGCAATCTATGGTTTTGTGCGCAGCCCTTAATGAAGGCAGGAAGTATGTG<br>GTTACATCAAAACAATTCCCATACATTAGTGAGTTGATTGAGCTTGGTGTGTTGAACAAAACTTTTTCCCGATG<br>GAATGGAAAGCATATATTATTCCCTATTGAGGATATTTACTGGACTGAATTAGTTGCCAGCTATGATCCATATA<br>ATATTGAGATAAAGCCAAGGCCAATATCTAAGTAACTAGATAAGAGGAATCGATTTTCCCTTAATTTTCTGGCG<br>TCCACTGCATGTTATGCCGCGTTCGCCAGGCTTGCTGTACCATGTGCGCTGATTCTTGCGCTCAATACGTTGCA<br>GGTTGCTTTCAATCTGTTTGTGGTATTCAGCCAGCACTGTAAGGTCTATCGGATTTAGTGCGCTTTCTACTCGT<br>GATTTCGGTTTGCGATTCAGCGAGAGAATAGGGCGGTTAACTGGTTTTGCGCTTACCCCAACCAACAGGGGATT<br>TGCTGCTTTCCATTGAGCCTGTTTCTCTGCGCGACGTTCGCGGCGGCGTGTTTGTGCATCCATCTGGATTCTCC<br>TGTCAGTTAGCTTTGGTGGTGTGTGGCAGTTGTAGTCCTGAACGAAAACCCCCCGCGATTGGCACATTGGCAGC<br>TAATCCGGAATCGCACTTACGGCCAATGCTTCGTTTCGTATCACACACCCCAAAGCCTTCTGCTTTGAATGCTG<br>CCCTTCTTCAGGGCTTAATTTTTAAGAGCGTCACCTTCATGGTGGTCAGTGCGTCCTGCTGATGTGCTCAGTAT<br>CACCGCCAGTGGTATTTATGTCAACACCGCCAGAGATAATTTATCACCGCAGATGGTTATCTGTATGTTTTTTA<br>TATGAATTTATTTTTTGCAGGGGGGCATTGTTTGGTAGGTGAGAGATCTGAATTGCTATGTTTAGTGAGTTGTA<br>TCTATTTATTTTTCAATAAATACAATTGGTTATGTGTTTTGGGGGCGATCGTGAGGCAAAGAAAACCCGGCGCT<br>GAGGCCGGGTTATTCTTGTTCTCTGGTCAAATTATATAGTTGGAAAACAAGGATGCATATATGAATGAACGATG<br>CAGAGGCAATGCCGATGGCGATAGTGGGTATCATGTAGCCGCTTATGCTGGAAAGAAGCAATAACCCGCAGAAA<br>AACAAAGCTCCAAGCTCAACAAAACTAAGGGCATAGACAATAACTACCGATGTCATATACCCATACTCTCTAAT<br>CTTGGCCAGTCGGCGCGTTCTGCTTCCGATTAGAAACGTCAAGGCAGCAATCAGGATTGCAATCATGGTTCCTG<br>CATATGATGACAATGTCGCCCCAAGACCATCTCTATGAGCTGAAAAAGAAACACCAGGAATGTAGTGGCGGAAA<br>AGGAGATAGCAAATGCTTACGATAACGTAAGGAATTATTACTATGTAAACACCAGGCATGATTCTGTTCCGCAT<br>AATTACTCCTGATAATTAATCCTTAACTTTGCCCACCTGCCTTTTAAAACATTCCAGTATATCACTTTTCATTC<br>TTGCGTAGCAATATGCCATCTCTTCAGCTATCTCAGCATTGGTGACCTTGTTCAGAGGCGCTGAGAGATGGCCT<br>TTTTCTGATAGATAATGTTCTGTTAAAATATCTCCGGCCTCATCTTTTGCCCGCAGGCTAATGTCTGAAAATTG<br>AGGTGACGGGTTAAAAATAATATCCTTGGCAACCTTTTTTATATCCCTTTTAAATTTTGGCTTAATGACTATAT<br>CCAATGAGTCAAAAAGCTCCCCTTCAATATCTGTTGCCCCTAAGACCTTTAATATATCGCCAAATACAGGTAGC<br>TTGGCTTCTACCTTCACCGTTGTTCGGCCGATGAAATGCATATGCATAACATCGTCTTTGGTGGTTCCCCTCAT<br>CAGTGGCTCTATCTGAACGCGCTCTCCACTGCTTAATGACATTCCTTTCCCGATTAAAAAATCTGTCAGATCGG<br>ATGTGGTCGGCCCGAAAACAGTTCTGGCAAAACCAATGGTGTCGCCTTCAACAAACAAAAAAGATGGGAATCCC<br>AATGATTCGTCATCTGCGAGGCTGTTCTTAATATCTTCAACTGAAGCTTTAGAGCGATTTATCTTCTGAACCAG<br>ACTCTTGTCATTTGTTTTGGTAAAGAGAAAAGTTTTTCCATCGATTTTATGAATATACAAATAATTGGAGCCAA<br>CCTGCAGGTGATGATTATCAGCCAGCAGAGAATTAAGGAAAACAGACAGGTTTATTGAGCGCTTATCTTTCCCT<br>TTATTTTTGCTGCGGTAAGTCGCATAAAAACCATTCTTCATAATTCAATCCATTTACTATGTTATGTTCTGAGG<br>GGAGTGAAAATTCCCCTAATTCGATGAAGATTCTTGCTCAATTGTTATCAGCTATGCGCCGACCAGAACACCTT<br>GCCGATCAGCCAAACGTCTCTTCAGGCCACTGACTAGCGATAACTTTCCCCACAACGGAACAACTCTCATTGCA<br>TGGGATCATTGGGTACTGTGGGTTTAGTGGTTGTAAAAACACCTGACCGCTATCCCTGATCAGTTTCTTGAAGG<br>TAAACTCATCACCCCCAAGTCTGGCTATGCAGAAATCACCTGGCTCAACAGCCTGCTCAGGGTCAACGAGAATT<br>AACATTCCGTCAGGAAAGCTTGGCTTGGAGCCTGTTGGTGCGGTCATGGAATTACCTTCAACCTCAAGCCAGAA<br>TGCAGAATCACTGGCTTTTTTGGTTGTGCTTACCCATCTCTCCGCATCACCTTTGGTAAAGGTTCTAAGCTTAG<br>GTGAGAACATCCCTGCCTGAACATGAGAAAAAACAGGGTACTCATACTCACTTCTAAGTGACGGCTGCATACTA<br>ACCGCTTCATACATCTCGTAGATTTCTCTGGCGATTGAAGGGCTAAATTCTTCAACGCTAACTTTGAGAATTTT<br>TGTAAGCAATGCGGCGTTATAAGCATTTAATGCATTGATGCCATTAAATAAAGCACCAACGCCTGACTGCCCCA<br>TCCCCATCTTGTCTGCGACAGATTCCTGGGATAAGCCAAGTTCATTTTTCTTTTTTTCATAAATTGCTTTAAGG<br>CGACGTGCGTCCTCAAGCTGCTCTTGTGTTAATGGTTTCTTTTTTGTGCTCATACGTTAAATCTATCACCGCAA<br>GGGATAAATATCTAACACCGTGCGTGTTGACTATTTTACCTCTGGCGGTGATAATGGTTGCATGTACTAAGGAG<br>GTTGTATGGAACAACGCATAACCCTGAAAGATTATGCAATGCGCTTTGGGCAAACCAAGACAGCTAAAGATCTC<br>GGCGTATATCAAAGCGCGATCAACAAGGCCATTCATGCAGGCCGAAAGATTTTTTTAACTATAAACGCTGATGG<br>AAGCGTTTATGCGGAAGAGGTAAAGCCCTTCCCGAGTAACAAAAAAACAACAGCATAAATAACCCCGCTCTTAC<br>ACATTCCAGCCCTGAAAAAGGGCATCAAATTAAACCACACCTATGGTGTATGCATTTATTTGCATACATTCAAT<br>CAATTGTTATCTAAGGAAATACTTACATATGGTTCGTGCAAACAAACGCAACGAGGCTCTACGAATCGAGAGTG<br>CGTTGCTTAACAAAATCGCAATGCTTGGAACTGAGAAGACAGCGGAAGCTGTGGGCGTTGATAAGTCGCGAATT<br>CGTCGTAGAATTCAACGTGGAATTCCTATCGGAATTCTCGGATGAATTCGTCGAGCCTCAGCTCATGTCATCCT<br>CAGCACACTTGACCCTCAGCTCAGCTAGCCTCAGCCTACAATCACCTCAGCGAATTCGGTGACCCTTACGCGAA<br>TCCGCTTTCAGACGTTGACTGGTCGCGTCTGGCAAAAGTTAAAGACCTGACGCCCGGCGAACTGACCGCTGAGT<br>CCTATGACGACAGCTATCTCGATGATGAAGATGCAGACTGGACTGCGACCGGGCAGGGGCAGAAATCTGCCGGA<br>GATACCAGCTTCACGCTGGCGTGGATGCCCGGAGAGCAGGGGCAGCAGGCGCTGCTGGCGTGGTTTAATGAAGG

	CGATACCCGTGCCTATAAAATCCGCTTCCCGAACGGCACGGTCGATGTGTTCCGTGGCTGGGTCAGCAGTATCG<br>GTAAGGCGGTGACGGCGAAGGAAGTGATCACCCGCACGGTGAAAGTCACCAATGTGGGACGTCCGTCGATGGCA<br>GAAGATCGCAGCACGGTAACAGCGGCAACCGGCATGACCGTGACGCCTGCCAGCACCTCGGTGGTGAAAGGGCA<br>GAGCACCACGCTGACCGTGGCCTTCCAGCCGGAGGGCGTAACCGACAAGAGCTTTCGTGCGGTGTCTGCGGATA<br>AAACAAAAGCCACCGTGTCGGTCAGTGGTATGACCATCACCGTGAACGGCGTTGCTGCAGGCAAGGTCAACATT<br>CCGGTTGTATCCGGTAATGGTGAGTTTGCTGCGGTTGCAGAAATTACCGTCACCGCCAGTTAATCCGGAGAGTC<br>AGCGATGTTCCTGAAAACCGAATCATTTGAACATAACGGTGTGACCGTCACGCTTTCTGAACTGTCAGCCCTGC<br>AGCGCATTGAGCATCTCGCCCTGATGAAACGGCAGGCAGAACAGGCGGAGTCAGACAGCAACCGGAAGTTTACT<br>GTGGAAGACGCCATCAGAACCGGCGCGTTTCTGGTGGCGATGTCCCTGTGGCATAACCATCCGCAGAAGACGCA<br>GATGCCGTCCATGAATGAAGCCGTTAAACAGATTGAGCAGGAAGTGCTTACCACCTGGCCCACGGAGGCAATTT<br>CTCATGCTGAAAACGTGGTGTACCGGCTGTCTGGTATGTATGAGTTTGTGGTGAATAATGCCCCTGAACAGACA<br>GAGGACGCCGGGCCCGCAGAGCCTGTTTCTGCGGGAAAGTGTTCGACGGTGAGCTGAGTTTTGCCCTGAAACTG<br>GCGCGTGAGATGGGGCGACCCGACTGGCGTGCCATGCTTGCCGGGATGTCATCCACGGAGTATGCCGACTGGCA<br>CCGCTTTTACAGTACCCATTATTTTCATGATGTTCTGCTGGATATGCACTTTTCCGGGCTGACGTACACCGTGC<br>TCAGCCTGTTTTTCAGCGATCCGGATATGCATCCGCTGGATTTCAGTCTGCTGAACCGGCGCGAGGCTGACGTC<br>GACAAGCTTCCGGCCGCAGTCGAGCACCACCACCACCACCACTGAGATCCGGCTGCTAACAAAGCCCGAAAGGA<br>AGCTGAGTTGGCTGCTGCCACCGCTGAGCAATAACTAGCATAACCCCTTGGGGCCTCTAAACGGGTCTTGAGGG<br>GTTTTTTGCTGAAAGGAGGAACTATATCCGGATTGGCGAATGGGACGCGCCCTGTAGCGGCGCATTAAGCGCGG<br>CGGGTGTGGTGGTTACGCGCAGCGTGACCGCTACACTTGCCAGCGCCCTAGCGCCCGCTCCTTTCGCTTTCTTC<br>CCTTCCTTTCTCGCCACGTTCGCCGGCTTTCCCCGTCAAGCTCTAAATCGGGGGCTCCCTTTAGGGTTCCGATT<br>TAGTGCTTTACGGCACCTCGACCCCAAAAAACTTGATTAGGGTGATGGTTCACGTAGTGGGCCATCGCCCTGAT<br>AGACGGTTTTTCGCCCTTTGACGTTGGAGTCCACGTTCTTTAATAGTGGACTCTTGTTCCAAACTGGAACAACA<br>CTCAACCCTATCTCGGTCTATTCTTTTGATTTATAAGGGATTTTGCCGATTTCGGCCTATTGGTTAAAAAATGA<br>GCTGATTTAACAAAAATTTAACGCGAATTTTAACAAAATATTAACGCTTACAATTTAGGTGGCACTTTTCGGGG<br>AAATGTGCGCGGAACCCCTATTTGTTTATTTTTCTAAATACATTCAAATATGTATCCGCTCATGAATTAATTCT<br>TAGAAAAACTCATCGAGCATCAAATGAAACTGCAATTTATTCATATCAGGATTATCAATACCATATTTTTGAAA<br>AAGCCGTTTCTGTAATGAAGGAGAAAACTCACCGAGGCAGTTCCATAGGATGGCAAGATCCTGGTATCGGTCTG<br>CGATTCCGACTCGTCCAACATCAATACAACCTATTAATTTCCCCTCGTCAAAAATAAGGTTATCAAGTGAGAAA<br>TCACCATGAGTGACGACTGAATCCGGTGAGAATGGCAAAAGTTTATGCATTTCTTTCCAGACTTGTTCAACAGG<br>CCAGCCATTACGCTCGTCATCAAAATCACTCGCATCAACCAAACCGTTATTCATTCGTGATTGCGCCTGAGCGA<br>GACGAAATACGCGATCGCTGTTAAAAGGACAATTACAAACAGGAATCGAATGCAACCGGCGCAGGAACACTGCC<br>AGCGCATCAACAATATTTTCACCTGAATCAGGATATTCTTCTAATACCTGGAATGCTGTTTTCCCGGGGATCGC<br>AGTGGTGAGTAACCATGCATCATCAGGAGTACGGATAAAATGCTTGATGGTCGGAAGAGGCATAAATTCCGTCA<br>GCCAGTTTAGTCTGACCATCTCATCTGTAACATCATTGGCAACGCTACCTTTGCCATGTTTCAGAAACAACTCT<br>GGCGCATCGGGCTTCCCATACAATCGATAGATTGTCGCACCTGATTGCCCGACATTATCGCGAGCCCATTTATA<br>CCCATATAAATCAGCATCCATGTTGGAATTTAATCGCGGCCTAGAGCAAGACGTTTCCCGTTGAATATGGCTCA<br>TAACACCCCTTGTATTACTGTTTATGTAAGCAGACAGTTTTATTGTTCATGACCAAAATCCCTTAACGTGAGTT<br>TTCGTTCCACTGAGCGTCAGACCCCGTAGAAAAGATCAAAGGATCTTCTTGAGATCCTTTTTTTCTGCGCGTAA<br>TCTGCTGCTTGCAAACAAAAAAACCACCGCTACCAGCGGTGGTTTGTTTGCCGGATCAAGAGCTACCAACTCTT<br>TTTCCGAAGGTAACTGGCTTCAGCAGAGCGCAGATACCAAATACTGTCCTTCTAGTGTAGCCGTAGTTAGGCCA<br>CCACTTCAAGAACTCTGTAGCACCGCCTACATACCTCGCTCTGCTAATCCTGTTACCAGTGGCTGCTGCCAGTG<br>GCGATAAGTCGTGTCTTACCGGGTTGGACTCAAGACGATAGTTACCGGATAAGGCGCAGCGGTCGGGCTGAACG<br>GGGGGTTCGTGCACACAGCCCAGCTTGGAGCGAACGACCTACACCGAACTGAGATACCTACAGCGTGAGCTATG<br>AGAAAGCGCCACGCTTCCCGAAGGGAGAAAGGCGGACAGGTATCCGGTAAGCGGCAGGGTCGGAACAGGAGAGC<br>GCACGAGGGAGCTTCCAGGGGGAAACGCCTGGTATCTTTATAGTCCTGTCGGGTTTCGCCACCTCTGACTTGAG<br>CGTCGATTTTTGTGATGCTCGTCAGGGGGGCGGAGCCTATGGAAAAACGCCAGCAACGCGGCCTTTTTACGGTT<br>CCTGGCCTTTTGCTGGCCTTTTGCTCACATGTTCTTTCCTGCGTTATCCCCTGATTCTGTGGATAACCGTATTA<br>CCGCCTTTGAGTGAGCTGATACCGCTCGCCGCAGCCGAACGACCGAGCGCAGCGAGTCAGTGAGCGAGGAAGCG<br>GAAGAGCGCCTGATGCGGTATTTTCTCCTTACGCATCTGTGCGGTATTTCACACCGCATATATGGTGCACTCTC<br>AGTACAATCTGCTCTGATGCCGCATAGTTAAGCCAGTATACACTCCGCTATCGCTACGTGACTGGGTCATGGCT<br>GCGCCCCGACACCCGCCAACACCCGCTGACGCGCCCTGACGGGCTTGTCTGCTCCCGGCATCCGCTTACAGACA<br>AGCTGTGACCGTCTCCGGGAGCTGCATGTGTCAGAGGTTTTCACCGTCATCACCGAAACGCGCGAGGCAGCTGC<br>GGTAAAGCTCATCAGCGTGGTCGTGAAGCGATTCACAGATGTCTGCCTGTTCATCCGCGTCCAGCTCGTTGAGT<br>TTCTCCAGAAGCGTTAATGTCTGGCTTCTGATAAAGCGGGCCATGTTAAGGGCGGTTTTTTCCTGTTTGGTCAC<br>TGATGCCTCCGTGTAAGGGGGATTTCTGTTCATGGGGGTAATGATACCGATGAAACGAGAGAGGATGCTCACGA<br>TACGGGTTACTGATGATGAACATGCCCGGTTACTGGAACGTTGTGAGGGTAAACAACTGGCGGTATGGATGCGG<br>CGGGACCAGAGAAAAATCACTCAGGGTCAATGCCAGCGCTTCGTTAATACAGATGTAGGTGTTCCACAGGGTAG<br>CCAGCAGCATCCTGCGATGCAGATCCGGAACATAATGGTGCAGGGCGCTGACTTCCGCGTTTCCAGACTTTACG<br>AAACACGGAAACCGAAGACCATTCATGTTGTTGCTCAGGTCGCAGACGTTTTGCAGCAGCAGTCGCTTCACGTT

CGCTCGCGTATCGGTGATTCATTCTGCTAACCAGTAAGGCAACCCCGCCAGCCTAGCCGGGTCCTCAACGACAG
GAGCACGATCATGCGCACCCGTGGGGCCGCCATGCCGGCGCCTGCCACCATACCCACGCCGAAACAAGCGCTCA
TGAGCCCGAAGTGGCGAGCCCGATCTTCCCCATCGGTGATGTCGGCGATATAGGCGCCAGCAACCGCACCTGTG
GCGCCGGTGATGCCGGCCACGATGCGTCCGGCGTAGAGGATCGAGATCTCGATCCCGCGAAATTAATACGACTC
ACTATAGGGGAATTGTGAGCGGATAACAATTCCCTTCTAGCCATATTGGACTCGGACCTGTTTCACGTGGAACA
CTGAGCCTGGACTAGGTCTAGCATATTGGACTCGACTTTCACGTGGAACACTGAGCCTGGACTAGGTCTAGAAC
AGGTTCTTTTTTCTTTGTTTCACGTGGAACATTCTGATTAATGTACAGCTAGCCATATTGGACTCGGACCTGTT
TCACGTGGAACACTGAGCCTGGACTAGGTCTAGAACAGGTTCTTTTTTCTTTGTTTCACGTGGAACATTCTGAT
TAATGTACAGCTAGAACAGGTTCTTTTTTCTTTGTTTCACGTGGAACATTCTGATTAATGTACAGCTAGAAATA
ATTTTGTTTAACTTTAAGAAGGAGATATACCATGTGTTTCACGTGGAACACATGGCTAAAGGCCTTGGAAAAGG
GATTAATGCGTTATTTAATCAGGTAGATTTGTCTGAAGAGACAGTTGAAGAAATTAAAATTGCCGATTTACGCC
CTAATCCTTATCAGCCAAGAAAACACTTTGATGACGAGGCATTAGCTGAACTAAAAGAATCTGTGCTGCAGCAT
GGCATTCTTCAGCCGCTTATCGTCAGAAAATCTTTAAAAGGCTATGATATTGTTGCGGGTGAACGGCGTTTTCG
AGCGGCAAAGCTGGCAGGTTTAGATACAGTTCCGGCCATTGTCCGTGAATTATCAGAGGCGTTAATGAGGGAAA
TTGCTTTATTAGAAAACCTTCAGCGTGAAGATTTATCTCCGCTTGAAGAGGCTCAGGCATATGCCGAAACAAGC
GCTCATGAGCCCGAAGTGGCGAGCCCGATCTTCCCCATCGGTGATGTCGGCGATATAGGCGCCAGCAACCGCAC
CTGTGGCGCCGGTGATGCCGGCCACGATGCGTCCGGCGTAGAGGATCGAGATCTCGATCCCGCGAAATTAATAC
GACTCACTATAGGGGAATTGTGAGCGGATAACAATTCCCTTCTAGCCATATTGGACTCGGACCTGTTTCACGTG
GAACACTGAGCCTGGACTAGGTCTAGCATATTGGACTCGACTTTCACGTGGAACACTGAGCCTGGACTAGGTCT
AGAACAGGTTCTTTTTTCTTTGTTTCACGTGGAACATTCTGATTAATGTACAGCTAGCCATATTGGACTCGGAC
CTGTTTCACGTGGAACACTGAGCCTGGACTAGGTCTAGAACAGGTTCTTTTTTCTTTGTTTCACGTGGAACATT
CTGATTAATGTACAGCTAGAACAGGTTCTTTTTTCTTTGTTTCACGTGGAACATTCTGATTAATGTACAGCTAG
AAATAATTTTGTTTAACTTTAAGAAGGAGATATACCATGTGTTTCACGTGGAACACATGGCTAAAGGCCTTGGA
AAAGGGATTAATGCGCATATGACTCCCTTTTGAAACACTTAGATCTCACACAAGAGCAGCTTGCCAAACGTCTT
GGGAAAAGCAGACCGCATATTGCGAATCATTTAAGACTGCTGACACTGCCAGAAAATATTCAACAGCTTATTGC
CGAAGGCACGCTTTCTATGGGACATGGACGCACGCTTCTTGGCTTAAAAAACAAAAATAAGCTTGAACCGCTGG
TACAAAAAGTGATTGCGGAGCAGCTCAATGTTCGCCAACTTGAGCAGCTGATTCAGCAGTTGAATCAGAATGTT
CCACGTGAAACAAAGAAAAAAGAACCTGTGAAAGATGCGGTTCGCCATGCCGGCGCCTGCCACCATACCCACGC
CGAAACAAGCGCTCATGAGCCCGAAGTGGCGAGCCCGATCTTCCCCATCGGTGATGTCGGCGATATAGGCGCCA
GCAACCGCACCTGTGGCGCCGGTGATGCCGGCCACGATGCGTCCGGCGTAGAGGATCGAGATCTCGATCCCGCG
AAATTAATACGACTCACTATAGGGGAATTGTGAGCGGATAACAATTCCCTTCTAGCCATATTGGACTCGGACCT
GTTTCACGTGGAACACTGAGCCTGGACTAGGTCTAGCATATTGGACTCGACTTTCACGTGGAACACTGAGCCTG
GACTAGGTCTAGAACAGGTTCTTTTTTCTTTGTTTCACGTGGAACATTCTGATTAATGTACAGCTAGCCATATT
GGACTCGGACCTGTTTCACGTGGAACACTGAGCCTGGACTAGGTCTAGAACAGGTTCTTTTTTCTTTGTTTCAC
GTGGAACATTCTGATTAATGTACAGCTAGAACAGGTTCTTTTTTCTTTGTTTCACGTGGAACATTCTGATTAAT
GTACAGCTAGAAATAATTTTGTTTAACTTTAAGAAGGAGATATACCATGTGTTTCACGTGGAACACATGGCTAA
AGGCCTTGGAAAAGGGATTAATGCGTTATTTAATCAGGTAGATTTGTCTGAAGAGACAGTTGAAGAAATTAAAA
TTGCCGATTTACGCCCTAATCCTTATCAGCCAAGAAAACACTTTGATGACGAGGCATTAGCTGAACTAAAAGAA
TCTGTGCTGCAGCATGGCATTCTTCAGCCGCTTATCGTCAGAAAATCTTTAAAAGGCTATGATATTGTTGCGGG
TGAACGGCGTTTTCGAGCGGCAAAGCTGGCAGGTTTAGATACAGTTCCGGCCATTGTCCGTGAATTATCAGAGG
CGTTAATGAGGGAAATTGCTTTATTAGAAAACCTTCAGCGTGAAGATTTATCTCCGCTTGAAGAGGCTCAGGCA
TATGCCGAAACAAGCGCTCATGAGCCCGAAGTGGCGAGCCCGATCTTCCCCATCGGTGATGTCGGCGATATAGG
CGCCAGCAACCGCACCTGTGGCGCCGGTGATGCCGGCCACGATGCGTCCGGCGTAGAGGATCGAGATCTCGATC
CCGCGAAATTAATACGACTCACTATAGGGGAATTGTGAGCGGATAACAATTCCCTTCTAGCCATATTGGACTCG
GACCTGTTTCACGTGGAACACTGAGCCTGGACTAGGTCTAGCATATTGGACTCGACTTTCACGTGGAACACTGA
GCCTGGACTAGGTCTAGAACAGGTTCTTTTTTCTTTGTTTCACGTGGAACATTCTGATTAATGTACAGCTAGCC
ATATTGGACTCGGACCTGTTTCACGTGGAACACTGAGCCTGGACTAGGTCTAGAACAGGTTCTTTTTTCTTTGT
TTCACGTGGAACATTCTGATTAATGTACAGCTAGAACAGGTTCTTTTTTCTTTGTTTCACGTGGAACATTCTGA
TTAATGTACAGCTAGAAATAATTTTGTTTAACTTTAAGAAGGAGATATACCATGTGTTTCACGTGGAACACATG
GCTAAAGGCCTTGGAAAAGGGATTAATGCGCATATGACTCCCTTTTGAAACACTTAGATCTCACACAAGAGCAG
CTTGCCAAACGTCTTGGGAAAAGCAGACCGCATATTGCGAATCATTTAAGACTGCTGACACTGCCAGAAAATAT
TCAACAGCTTATTGCCGAAGGCACGCTTTCTATGGGACATGGACGCACGCTTCTTGGCTTAAAAAACAAAAATA
AGCTTGAACCGCTGGTACAAAAAGTGATTGCGGAGCAGCTCAATGTTCGCCAACTTGAGCAGCTGATTCAGCAG
TTGAATCAGAATGTTCCACGTGAAACAAAGAAAAAAGAACCTGTGAAAGATGCGGTTCGCCATGCCGGCGATAA
TGGCCTGCTTCTCGCCGAAACGTTTGGTGGCGGGACCAGTGACGAAGGCTTGAGCGAGGGCGTGCAAGATTCCG
AATACCGCAAGCGACAGGCCGATCATCGTCGCGCTCCAGCGAAAGCGGTCCTCGCCGAAAATGACCCAGAGCGC
TGCCGGCACCTGTCCTACGAGTTGCATGATAAAGAAGACAGTCATAAGTGCGGCGACGATAGTCATGCCCCGCG
CCCACCGGAAGGAGCTGACTGGGTTGAAGGCTCTCAAGGGCATCGGTCGAGATCCCGGTGCCTAATGAGTGAGC
TAACTTACATTAATTGCGTTGCGCTCACTGCCCGCTTTCCAGTCGGGAAACCTGTCGTGCCAGCTGCATTAATG

	AATCGGCCAACGCGCGGGGAGAGGCGGTTTGCGTATTGGGCGCCAGGGTGGTTTTTCTTTTCACCAGTGAGACG<br>GGCAACAGCTGATTGCCCTTCACCGCCTGGCCCTGAGAGAGTTGCAGCAAGCGGTCCACGCTGGTTTGCCCCAG<br>CAGGCGAAAATCCTGTTTGATGGTGGTTAACGGCGGGATATAACATGAGCTGTCTTCGGTATCGTCGTATCCCA<br>CTACCGAGATATCCGCACCAACGCGCAGCCCGGACTCGGTAATGG
C-Trap long dsDNA fragments after PCR for the loop-containing DNA<br>(7688 bp)	**GGCGGCGAGCTGA**GGGTTACCGGATAAGGCGCAGCGGTCGGGCTGAACGGGGGGTTCGTGCACACAGCCCAGCT<br>TGGAGCGAACGACCTACACCGAACTGAGATACCTACAGCGTGAGCTATGAGAAAGCGCCACGCTTCCCGAAGGG<br>AGAAAGGCGGACAGGTATCCGGTAAGCGGCAGGGTCGGAACAGGAGAGCGCACGAGGGAGCTTCCAGGGGGAAA<br>CGCCTGGTATCTTTATAGTCCTGTCGGGTTTCGCCACCTCTGACTTGAGCGTCGATTTTTGTGATGCTCGTCAG<br>GGGGGCGGAGCCTATGGAAAAACGCCAGCAACGCGGCCTTTTTACGGTTCCTGGCCTTTTGCTGGCCTTTTGCT<br>CACATGTTCTTTCCTGCGTTATCCCCTGATTCTGTGGATAACCGTATTACCGCCTTTGAGTGAGCTGATACCGC<br>TCGCCGCAGCCGAACGACCGAGCGCAGCGAGTCAGTGAGCGAGGAAGCGGAAGAGCGCCCAATACGCAAACCGC<br>CTCTCCCCGCGCGTTGGCCGATTCATTAATGCAGGTTAACCTGGCTTATCGAAATTAATACGACTCACTATAGG<br>GAGACCGGCCTCGAGCCATTTAAGGCGTTATCCCCAGTTTTTAGTGAGATCTCTCCCACTGACGTATCATTTGG<br>TCCGCCCGAAACAGGTTGGCCAGCGTGAATAACATCGCCAGTTGGTTATCGTTTTTCAGCAACCCCTTGTATCT<br>GGCTTTCACGAAGCCGAACTGTCGCTTGATGATGCGAAATGGGTGCTCCACCCTGGCCCGGATGCTGGCTTTCA<br>TGTATTCGATGTTGATGGCCGTTTTGTTCTTGCGTGGATGCTGTTTCAAGGTTCTTACCTTGCCGGGGCGCTCG<br>GCGATCAGCCAGTCCACATCCACCTCGGCCAGCTCCTCGCGCTGTGGCGCCCCTTGGTAGCCGGCATCGGCTGA<br>GACAAATTGCTCCTCTCCATGCAGCAGATTACCCAGCTGATTGAGGTCATGCTCGTTGGCCGCGGTGGTGACCA<br>GGCTGTGGGTCAGGCCACTCTTGGCATCGACACCAATGTGGGCCTTCATGCCAAAGTGCCACTGATTGCCTTTC<br>TTGGTCTGATGCATCTCCGGATCGCGTTGCTGCTCTTTGTTCTTGGTCGAGCTGGGTGCCTCAATGATGGTGGC<br>ATCGACCAAGGTGCCTTGAGTCATCATGACGCCTGCTTCGGCCAGCCAGCGATTGATGGTCTTGAACAATTGGC<br>GGGCCAGTTGATGCTGCTCCAGCAGGTGGCGGAAATTCATGATGGTGGTGCGGTCCGGCAAGGCGCTATCCAGG<br>GATAACCGGGCAAACAGACGCATGGAGGCGATTTCGTACAGAGCATCTTCCATCGCGCCATCGCTCAGGTTGTA<br>CCAATGCTGCATGCAGTGAATGCGTAGCATGGTTGCTAGCAGATAAGGTCGCCGGCCATTACCAGCCTTGGGGT<br>AAAACGGCTCGATGACTTCCACCATGTTTTGCCATGGCAGAATCTGCTCCATGCGGGACAAGAAAATCTCTTTT<br>CTGGTCTGACGGCGCTTACTGCTGAATTCACTGTCGGCGAAGGTAAGTTGATGACTCATGATGAACCCTGTTCT<br>ATGGCTCCAGATGACAAACATGATCTCATATCAGGGACTTGTTCGCACCTTCCTTAATCCCTAACATAAATGAC<br>ACAATAAAATCAGAAAGCACAGGAAATTTTCTTACGGAATAATCATGTAAAGATTTCGCTTCATCTCGGAACAG<br>GCATAATGTACTAAAAGCATAACGCTTACTTTCGACATATTTAATATATGTCAGCAAATCATCATGCTTCATGA<br>TGATATCGGGATTCAAAAACAAAATGTAATCATCATCTGCGGGTCTATATTTTTCCTTTACATACGCCACCGCA<br>ATATTATTATTATGACCAAAGCCGTATACACCTCCACTAATATAGTCCAGGCCTGCATAATGCTGGCATATTTG<br>TTTCAATAATAGAGAGTCTTTGTTGTCGCGTACGATAATCTTGTAGTGCTCATCGTCAGCATTAAGATTTTCGA<br>GTAATTTTTTGATGTAGTCTTCATGTCCGTGGGAAACGATTATTATATATACCATTTCAATGTTCTTCAGTAAT<br>AAAATTAACTAGTTCATCAAACCCAACTAATACATTTTCATTACGATAAATGAAATTTGCATCAGAGATATCTT<br>TTTTGAGGTTACCTTTTTTGAAGTCAATAATAAGTTTAACTAACATGTCATCGTTATTAGAATCAAAAAAAGCT<br>TTCTTTTCATAACTACCAAGAGTTTCTCTAGTAAATGGGAAATCTGATGCTAATACCCACTTACCTCGCTCTTT<br>AGCTTCAGACAACGGCAATCCCCATGTTTCTAACCTAGAGGGAAAACAAACTATATCTGAAATATTATAACAAT<br>GATCGATTTTTTCTTTATCCAAGTACCCGAGGAAATGAACATTATCCAGTCCTTCTGCAAGACTGATAATATAT<br>TTTGCATACGCATTTTCTGTACCACTGATAGTAAGCAGAAATTTAATATTGGATTGTTCTTTCAATTTCCTTGC<br>TGCACTAATAATAAGCTCGTAATTTTTAAATACTCGTGGAACAGCAGGGTAAAATATTGTCAACTCAGAAGGGT<br>TATTCTTAAATTGAGAATCATCATCAGTAAGTTGGCTTTTATCAGATAATTTAATTTCTGGCCGACTGACAATG<br>ATGTTATTTATAGAATATTTCTTGATAAATTTTTCTTTCATCCAGAATTGTTGAACAAACACTGCAGTATTTTT<br>TTTAATGTTTATTTTATATATCAGCCCGTATAGCATTTTAAATAAGAAAAAGCTAGGCTCCATAAGAATTTCAC<br>GGAATAAAATTCCTTTATAAAAAGGTGCAGGGTTATGACAATACACATATCTTTTTTTAGTGACGACATTGGCC<br>GTAATATCATGCAGACAAATCCAATGCGTAGCATTCAGCTCTTTTGAAAGTTTTTTACAAACTACATATTCAAA<br>GTGCAAACGTTTTAGCCACGACCCTTTAACCTCAGGAAACTCAATGAATTTAACCCATGGATAACTTTCTTTTA<br>ACTCTTTAGCAGAATGGACTAATGCGATAAAACTGACATTTTCTTTATTATTAGTTGCTGCCAAAAATTTTTTC<br>AAAATGGTAAATGGACCGCCAGTGGTAAAATTGACCGCAGAAACAACGACTATGCTTTTTCCCATAATTTGGTC<br>TCATGATTGTATTTCTTTATGATTTTTGCTGGTACTCCCGCAATGACAGTATTTTCGGGAATAGAACCTCTAAC<br>AACAGAATTGGCGCCGACTACGACTCCATTACCAATAATTGTTCCAGGCAAAACCGTCACATTCTCACCCAACC<br>AAACCCTCTGGCCAATTACAACAGCTGAAGATTCCAACGTGCGCATGTCTGGAGGTATATTTGGCGAACTCATT<br>GGATCAGAGTGCTTAAAGGAACCGTGATTATGATCGGTAATAAATACTTTACTTGCAATAAGCGTATCCCGACC<br>TATCGTAACGCTCTCAATTGAGGCGATATGAACATAGTCGTTAACTTGCACATTATCGGAAAAAAAAATCACGC<br>CACGTCCAAATGCATCCAGCCTGAGACCGACTCCACTTGTGAAATTTTCACCAAAATTAATGCTACCATCATTG<br>CGAATATAGCAGGGAAATCGAATAATTCTACAGTTCCGGTAAAATACACGAGTCAATAAGACATCTCTAACAAG<br>CCGAATAAAACCACAGAGACCATATCGTTTAGCGAGTTTTAAGATCATCGATCACCTCTTCAAGAACATCCCTG<br>AAGTAACTTCCTGTTCGAATTTTCTGAGAAATAATTTTTGTATTCTCACTAATTTGCTTATAAGTTTCTATTGT<br>CATGGAGTCAACAATCTCTTGCATTTCTTTGATTGATCCCACTGCATATCCTATTCTATTATCTACAATGAAAT

	CCGCAAGGGCGGCTTTATCCCATATAAATACTGGAAGTTCCATTGAAAGATAAAGAGATGTCTTATGAGGGTTA<br>TTAAACTTTAAATAGTCGCCAAAGGCACCACTACAGGTTTCGACAGAATCTCCATCCCAAATGAGTCCAAATTG<br>CATGCCTGGGAGGTTAATCTTTTCCGGAGATTGAGCATCAAAACTTCCAAGATATTTAGGATTATCTTTATTTT<br>CATAGTTGACACCAAAGAGAGTAAAATCGCATCCTTCAGTATATATGAAAGAACATTTATGCCTAGAAAGGTTG<br>CCAGCATATATGACCCCTCGTTGCTTATCCGTAACATCTCGATGCTCCACATCAGATGAGACGAGGTAATCAAA<br>TATTTTTATGTCTTTGATTTTATCCTGAGACATATATTTACTAAGGTACTTTGTCATTTGTGGATTGTGACTTA<br>TGACCATATCACAGGTAGCAAGCCGCACAGAATCACTACCCCCTCCTCCTCTTAATTCATCAATATCATGAATC<br>AGAGGTACTATTCTAAATTTTAGAAGGCGGTGAAAGAATGACAATATATGCCAAAATGGTTTGGCCATCGGGAA<br>ATTGAAAATTAAAACATCTTTATTTTCAAGACCGCAGAGAAATGTACTAAGCTTAACAGAACTAATAATTCTCT<br>GGACTACTCCACCCCATAGAGGAATGTTAACAACAGAAATGTTTTCATAATCTGAAGCAATGTCCAGTGCATCT<br>TTTCTTGCTTTAAATCCAGCATCGCGTCTAGAGAAATTTAAATCATTCAAAAAATACATTTTCACTTTATTTTC<br>TGGGCCTTAAGAAATTGAGAGAATACTATGATACAAAGAGTTATTTGTATCCAACTGCTAATATTAGTCATGAA<br>GCTTTCATGATAAAAAATGAAAGAAAAGGTATAAATAAAATATGAATAAAATATTTTCACAGATATGTAATTTC<br>GAGACAATCTCCATAAAACACCTGAAATACAGCCATGAATAACCATCATCAAATAGCTTAGCTCCGCGGAAATA<br>TAAACATAATCCGAAAAAGCAGTATAAACATTTGTTGGCAAACCCACCCACACAAATTCTTTATGCAACGACAT<br>AGAGACTCCACCTGTTAATAGCCCCATCAGCCTTTCAAAAAACCAAAAGACATGAGAACTGGCAGAGTTAGATA<br>CTTGCTGAAAATAAAACTCCTGAAACGCGATTATAGGGCTGACCAAATACATGGATAGATAATATGCCATCCCC<br>CCAGGCAGTCCACGTAAAAAGAGCATATACAAGGAGAATAGAACACCTACAGCTGTAATAAGATAAACATAATG<br>TTTTACTCTATTAACACCTACGATGAATGCATAAGAGATGATAACCATAAAGACAATTTGCTTACCAGTATTCA<br>GTATAATTGCAAAGATGAATACAATAAAAACAAGTAAAGTAAATGTTTTACTTACCTTTGTATTAGTAAATTTT<br>TTAGACCAAATAAATAAAGCAAAAGTAGTTAGAATGATTGGCTGCATGTATGCTGAGAAATTTCTTGATGTGTC<br>TTCAACATCAGCATCTCTTATCAAATTCATATAGCTAAGTAAGCTAGTCCCGAACTGGTAGTTACTTAACCTCA<br>TGCATATATATATCATCGAAAAAGAAATAACTAACAAGCCTACATTATGCACTTTCTTCGATGGTATGCTATAA<br>ATAGCATTATTGACTTTTCTGATATTTAGATCTAATACACTTTCCGTCAATAAACATGACAGGGTAAATGTCAA<br>AACATTGCAAAGTAGAAAAATCAACGTAGCGTCATTTAACTGAAAAGCATATATATCTGACGTTATTTCATATC<br>CCAATAAGACCAGTGCGAAGATGATATTAACGGATCCCCGGGTACCGAGCTCGAATTCATCGATGATATCAGAT<br>CTGGTTCTATAGTGTCACCTAAATCGTATGTGTATGATACATAAGGTTATGTATTAATTGTAGCCGCGTTCTAA<br>CGACAATATGTCCATATGGTGCACTCTCAGTACAATCTGCTCTGATGCCGCATAGTTAAGCCAGCCCCGACACC<br>CGCCAACACCCGCTGACGCGCCCTGACGGGCTTGTCTGCTCCCGGCATCCGCTTACAGACAAGCTGTGACCGTC<br>TCCGGGAGCTGCATGTGTCAGAGGTTTTCACCGTCATCACCGAAACGCGCGAGACGAAAGGGCCTCGTGATACG<br>CCTATTTTTATAGGTTAATGTCATGATAATAATGGTTTCTTAGACGTCAGGTGGCACTTTTCGGGGAAATGTGC<br>GCGGAACCCCTATTTGTTTATTTTTCTAAATACATTCAAATATGTATCCGCTCATGAGACAATAACCCTGATAA<br>ATGCTTCAATAATATTGAAAAAGGAAGAGTATGAGTATTCAACATTTCCGTGTCGCCCTTATTCCCTTTTTTGC<br>GGCATTTTGCCTTCCTGTTTTTGCTCACCCAGAAACGCTGGTGAAAGTAAAAGATGCTGAAGATCAGTTGGGTG<br>CACGAGTGGGTTACATCGAACTGGATCTCAACAGCGGTAAGATCCTTGAGAGTTTTCGCCCCGAAGAACGTTTT<br>CCAATGATGAGCACTTTTAAAGTTCTGCTATGTGGCGCGGTATTATCCCGTATTGACGCCGGGCAAGAGCAACT<br>CGGTCGCCGCATACACTATTCTCAGAATGACTTGGTTGAGTACTCACCAGTCACAGAAAAGCATCTTACGGATG<br>GCATGACAGTAAGAGAATTATGCAGTGCTGCCATAACCATGAGTGATAACACTGCGGCCAACTTACTTCTGACA<br>ACGATCGGAGGACCGAAGGAGCTAACCGCTTTTTTGCACAACATGGGGGATCATGTAACTCGCCTTGATCGTTG<br>GGAACCGGAGCTGAATGAAGCCATACCAAACGACGAGCGTGACACCACGATGCCTGTAGCAATGGCAACAACGT<br>TGCGCAAACTATTAACTGGCGAACTACTTACTCTAGCTTCCCGGCAACAATTAATAGACTGGATGGAGGCGGAT<br>AAAGTTGCAGGACCACTTCTGCGCTCGGCCCTTCCGGCTGGCTGGTTTATTGCTGATAAATCTGGAGCCGGTGA<br>GCGTGGGTCTCGCGGTATCATTGCAGCACTGGGGCCAGATGGTAAGCCCTCCCGTATCGTAGTTATCTACACGA<br>CGGGGAGTCAGGCAACTATGGATGAACGAAATAGACAGATCGCTGAGATAGGTGCCTCACTGATTAAGCATTGG<br>TAACTGTCAGACCAAGTTTACTCATATATACTTTAGATTGATTTAAAACTTCATTTTTAATTTAAAAGGATCTA<br>GGTGAAGATCCTTTTTGATAATCTCATGACCAAAATCCCTTAACGTGAGTTTTCGTTCCACTGAGCGTCAGACC<br>CCGTAGAAAAGATCAAAGGATCTTCTTGAGATCCTTTTTTTCTGCGCGTAATCTGCTGCTTGCAAACAAAAAAA<br>CCACCGCTACCAGCGGTGGTTTGTTTGCCGGATCAAGAGCTACCAACTCTTTTTCCGAAGGTAACTGGCTTCAG<br>CAGAGCGCAGATACCAAATACTGTTCTTCTAGTGTAGCCGTAGTTAGGCCACCACTTCAAGAACTCTGTAGCAC<br>CGCCTACATACCTCGCTCTGCTAATCCTGTTACCAGTGGCTGCTGCCAGGCTGA**GGGCATGCATGG**
ATTO488-dsDNA molecule<br>(139 bp)	CGACTCACTATAGGGAGACCGGCCTCGAGCCATTTAAGGCGTTATCCCCAGTTTTTAGTGAGATCTCTCCCACT<br>GACGTATCATTTGGTCCGCCCGAAACAGGTTGGCCAGCGTGAATAACATCGCCAGTTGGTTATCG

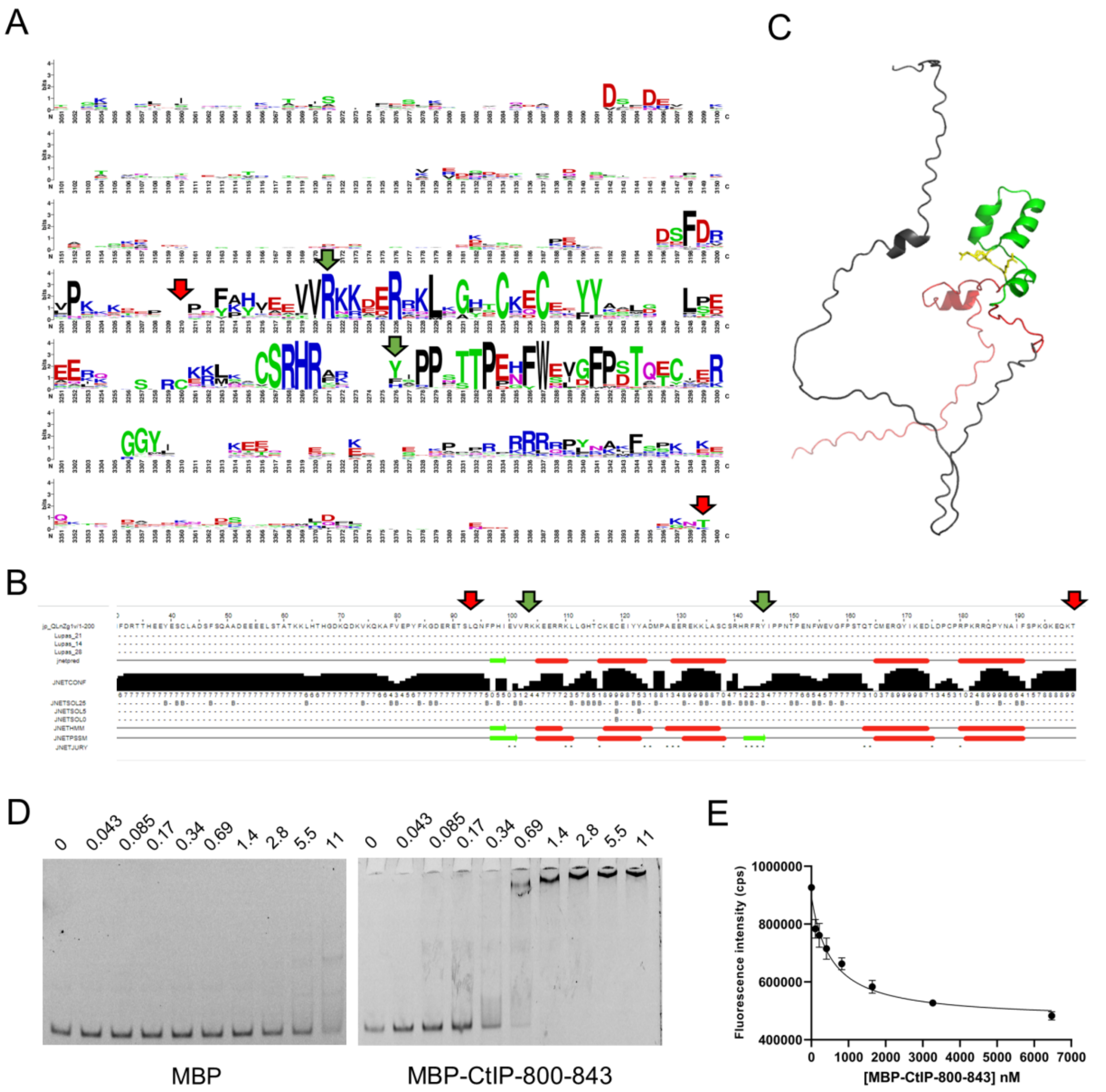

Supplementary Figure 1. Design of minimal DNA binding constructs ΔN790 and MBP-CtIP$^{800\text{-}843}$.

(A) Weblogo (6) format multiple sequence alignment of C-terminal region of diverse CtIP homologues. The red and green arrows indicate the start and end positions of the ΔN790 and MBP-CtIP-800-843 constructs respectively. (B) Secondary structure prediction for the final 200 amino acids of human CtIP (grey, coil; green, sheet; red, helix) (7). The red and green arrows indicate the start and end positions of the ΔN790 and MBP-CtIP-800-843 constructs respectively. (C) AlphaFold2 model of the final 200 amino acids of human CtIP (8). The N-terminal region of the construct is poorly ordered as expected from the secondary structure prediction. The better folded ribbons define the boundaries of the ΔN790 and

MBP-CtIP-800-843 constructs, which are better folded in the model, respectively. The amino acids of the conserved RHR motif which reside within the folded region are shown in yellow. (D) EMSA assays showing DNA binding by the MBP-CtIP-800-843 protein (but not by MBP alone). (E) DNA binding measured using MBP-CtIP-800-843 and the fluorescence quenching assay with a forked DNA substrate. The data were fit to a simple weak binding equation to yield Kd = 520 nM (see Methods for details).

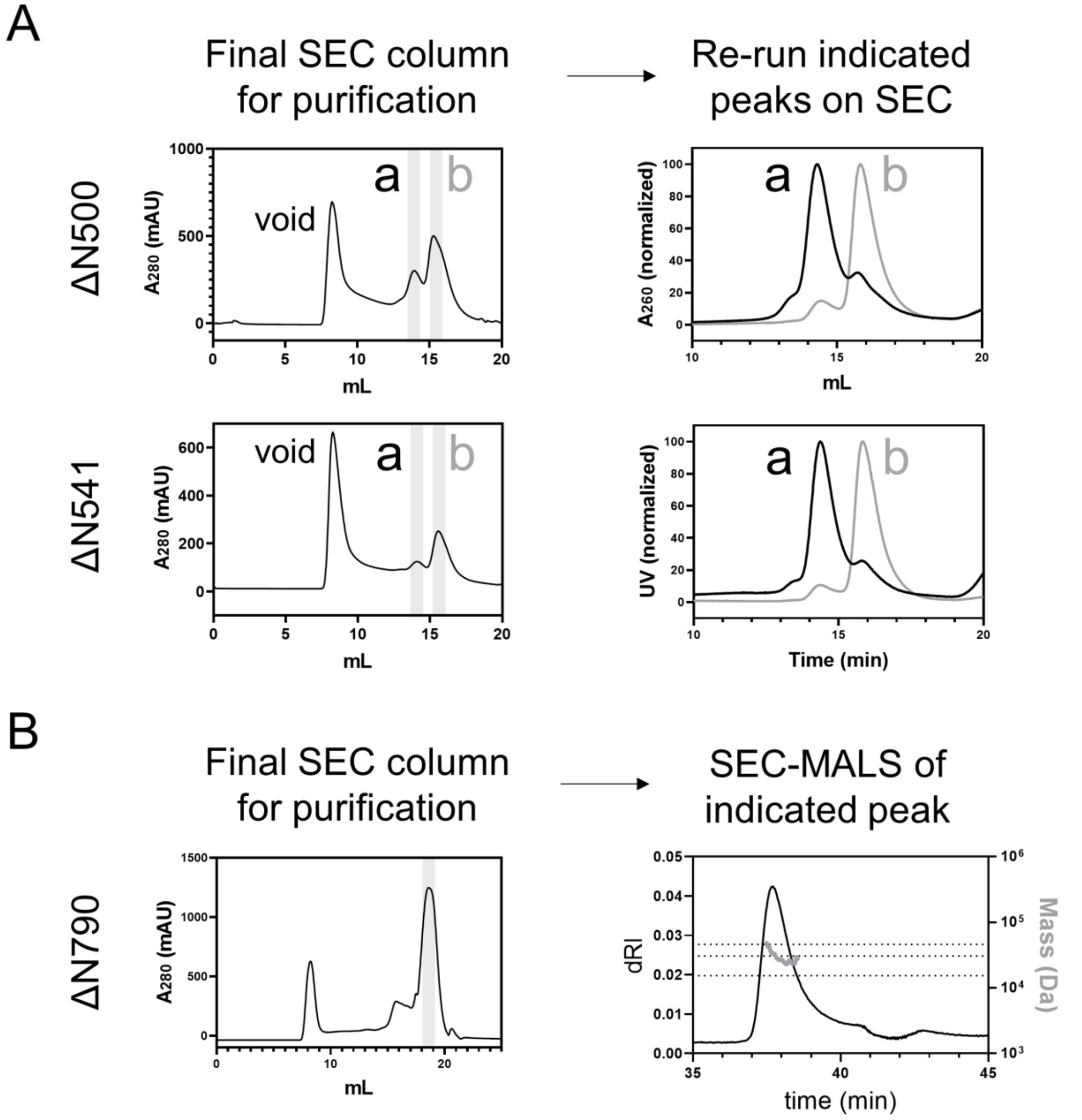

Supplementary Figure 2. SEC and SEC-MALS analyses of ΔN variants.

(A) The ΔN500 and ΔN541 variants both ran as two peaks (a and b) during the final size exclusion chromatography (SEC) run during purification. When selected peaks were re-run on SEC columns, they did not fully re-equilibrate into the two original peaks. In combination with MALS analysis (see main text) this suggests that these variants exist as a slowly exchanging population of monomers and dimers. (B) The ΔN790 variant was purified by SEC and then subjected to SEC-MALS analysis. The data quality was poor, yielding equivocal molecular weights ranging from 1.5 to 3.1x the value expected for a ΔN790 monomer. The dotted lines on the graph indicated the expected masses for a 1mer, 2mer and 3mer.

Therefore, this variant was also analysed by native mass spectrometry which showed it was monomeric (see main text).

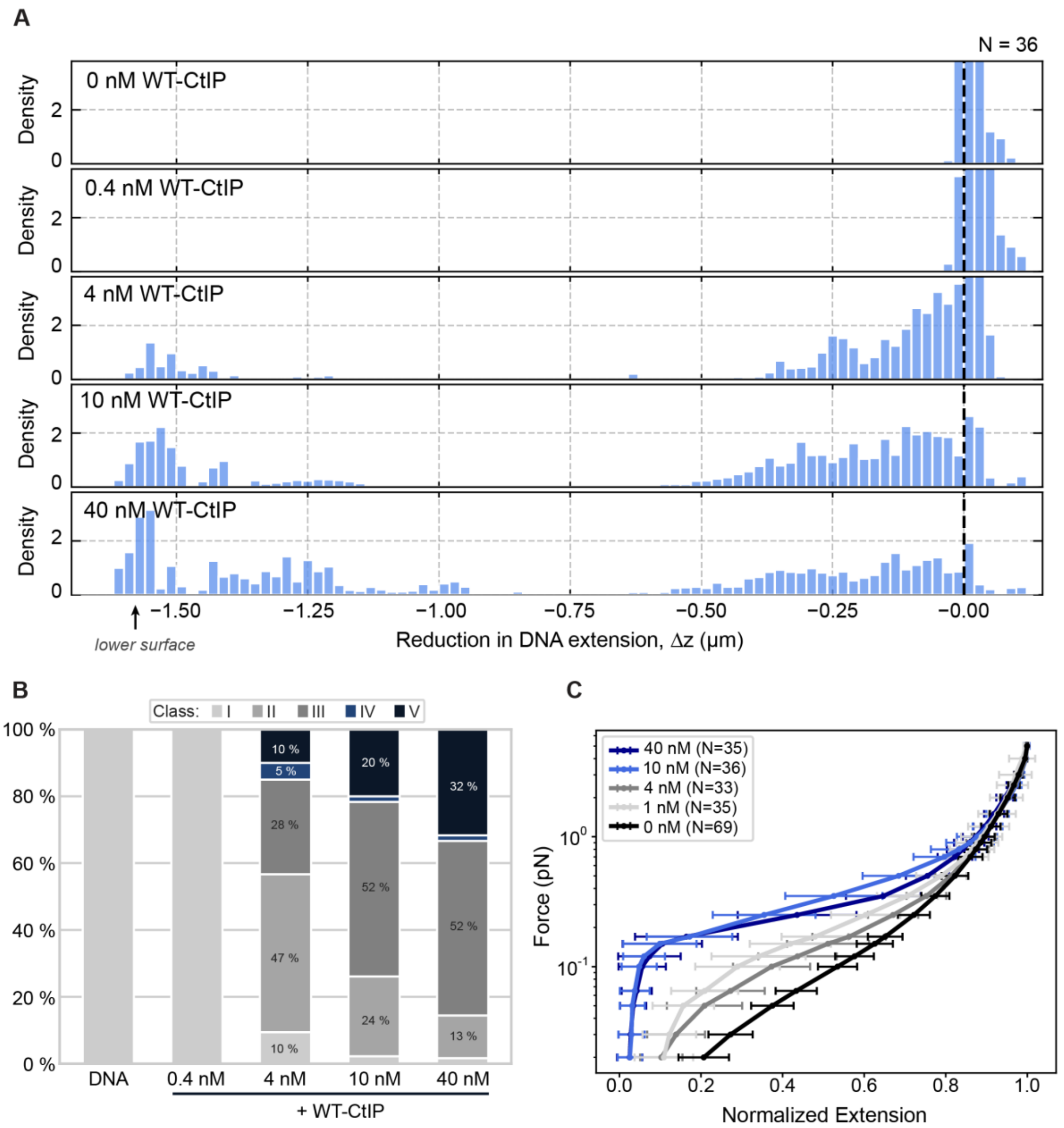

Supplementary Figure 3. CtIP bridging assay at different protein concentrations. (**A**) Distribution of the reduction in DNA extension (Δz) of a 4.3 kbp dsDNA in the presence of various WT-CtIP concentrations. The peak at Δz = 0 µm represents the extended states, while the peak at Δz = -1.6 µm represents complete condensation/attachment to the surface. (**B**) Classification of bridging events for increasing concentrations of WT-CtIP. (**C**) Force-extension curves with increasing concentration of WT-CtIP. Starting with the DNA molecules completely extended at a 5 pN force, the protein is flushed into the magnetic tweezers' microfluidic chamber. Then, for a period of 15 minutes the force is slowly reduced stepwise until completely removed. Assays were performed for 0, 1, 4, 10 and 40 nM [WT-CtIP]. N indicates the number of molecules studied for each condition.

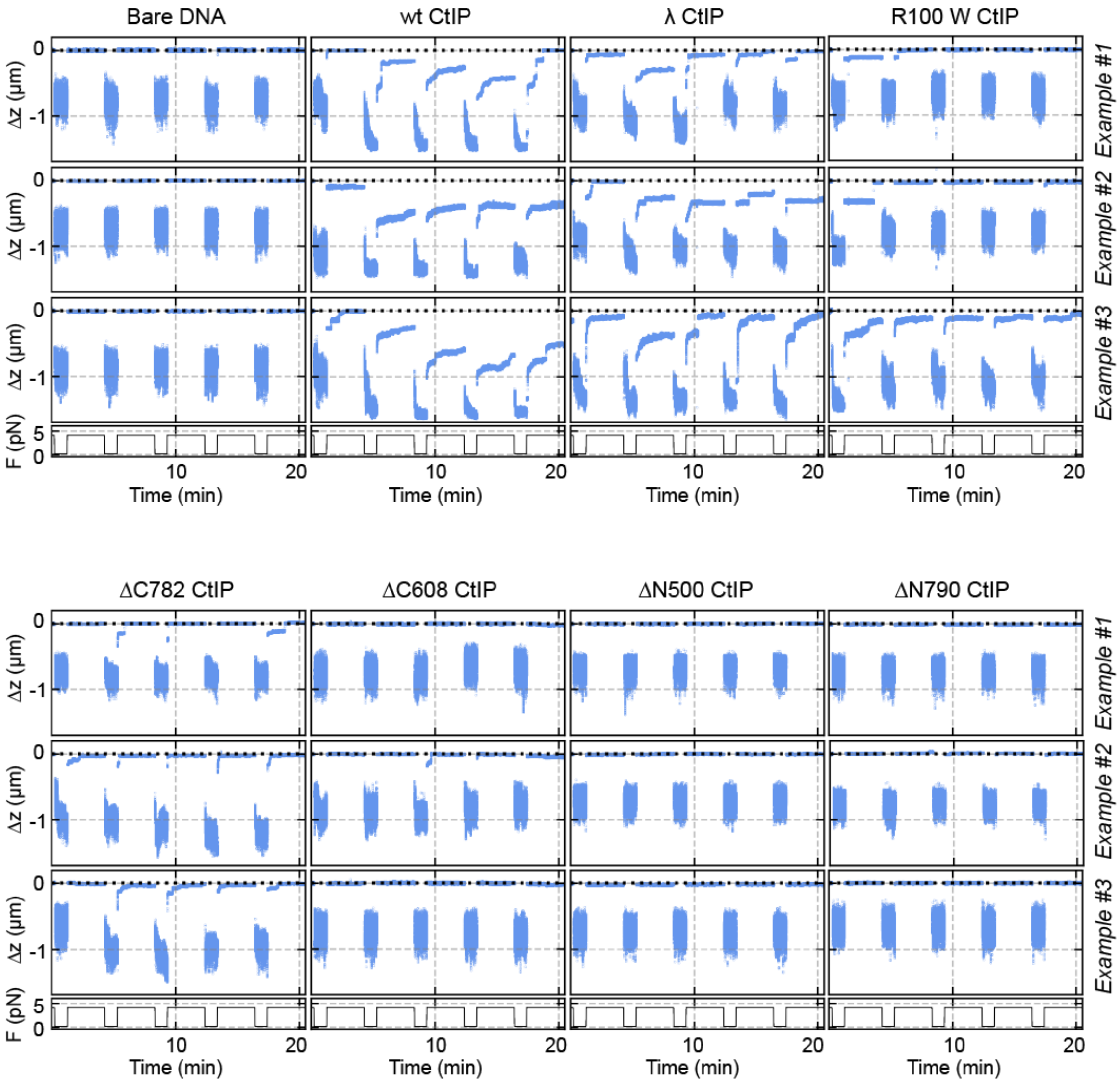

Supplementary Figure 4. Representative time-courses from the magnetic tweezers' experiments.

Individual time courses for the different wild-type and mutant CtIP variations studied in this work. See methods for a detailed description of the magnetic tweezers force-cycle assay.

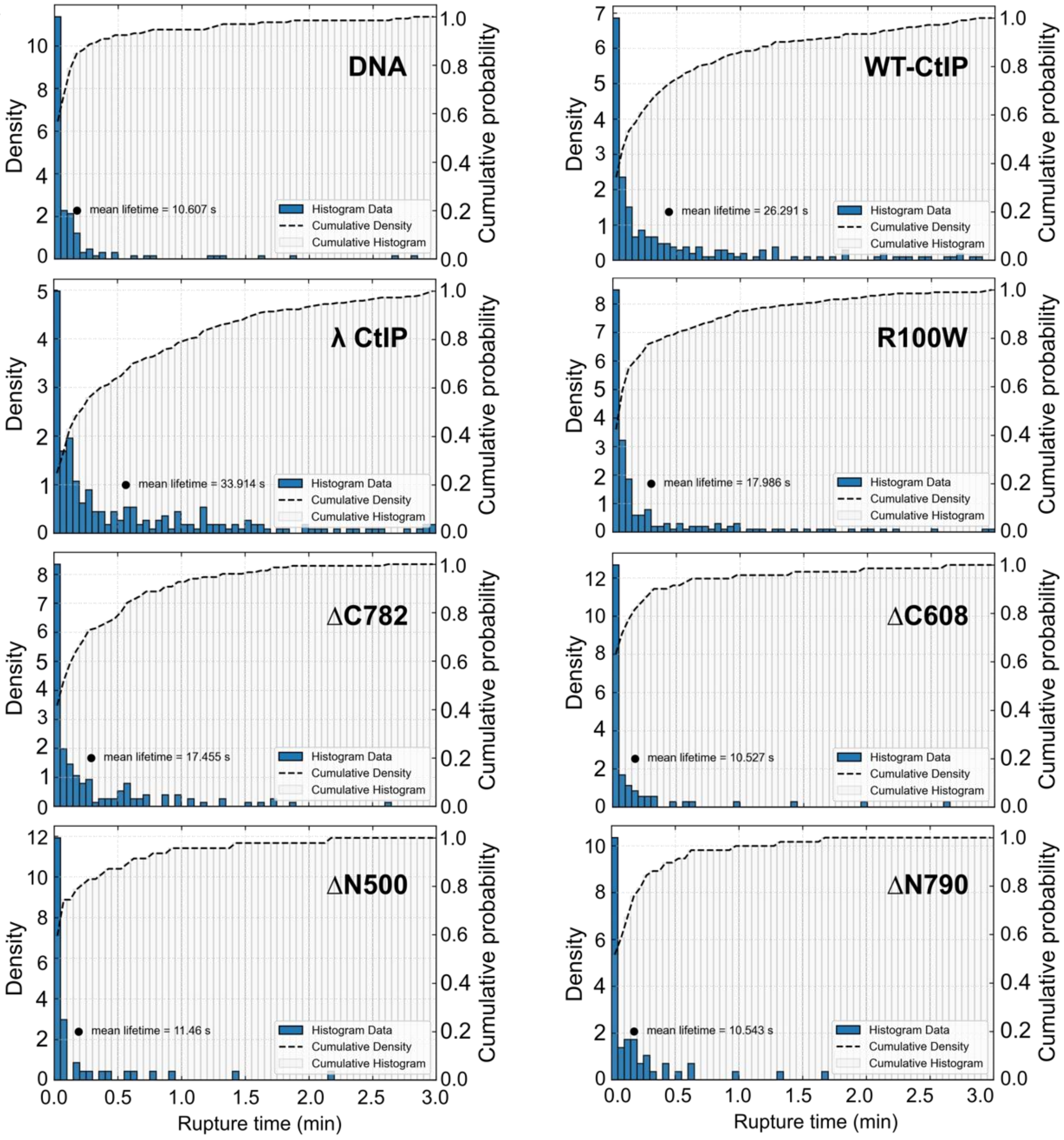

Supplementary Figure 5. Lifetime distributions for Class II events.

Each panel represents the distribution of the rupture time, the cumulative probability of rupture and the average lifetime before rupture for each CtIP variant studied.

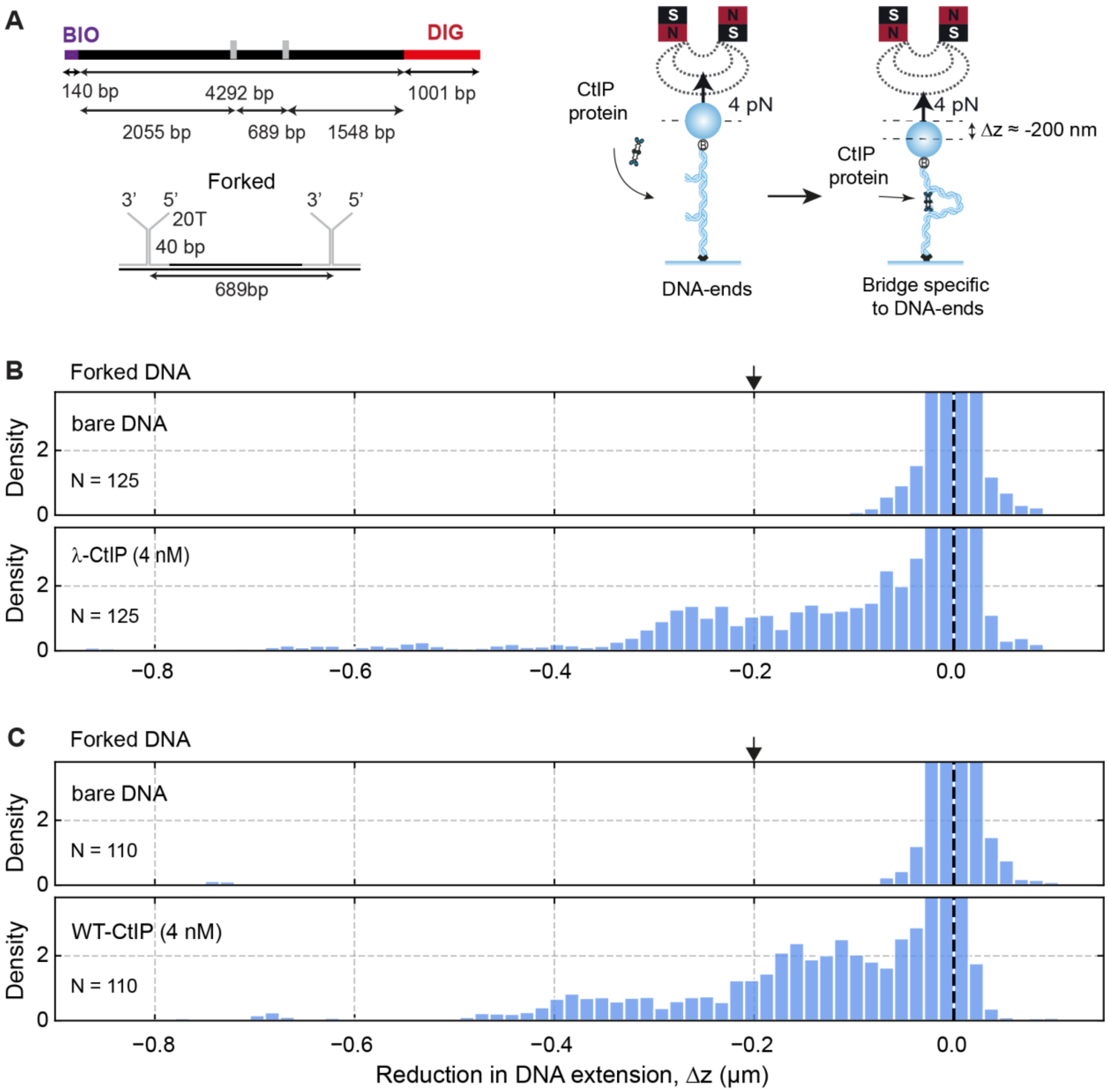

Supplementary Figure 6. Bridging interactions do not specifically tether DNA ends. (**A**) Structure of the branched DNA substrate with forked ends to study dsDNA bridging with MT and cartoon of the MT assay. A zoom of the branched region is shown (not to scale). (**B**) Histogram of the relative reductions (Δz) for DNA molecules containing two forked branches in the absence of protein (upper panel) and in the presence of 4 nM λ-CtIP (lower panel). (**C**) Histogram of the relative reductions (Δz) for DNA molecules containing two forked branches in the absence of protein (upper panel) and in the presence of 4nM WT-CtIP (lower panel). The peak at Δz = 0 μm represents all extended states and the densities for Δz < 0 μm represent all bridged states through CtIP interactions. Arrows indicate the expected

reduction for a specific end-to-end bridging between both fork ends. N indicates the number of DNA molecules studied for each condition.

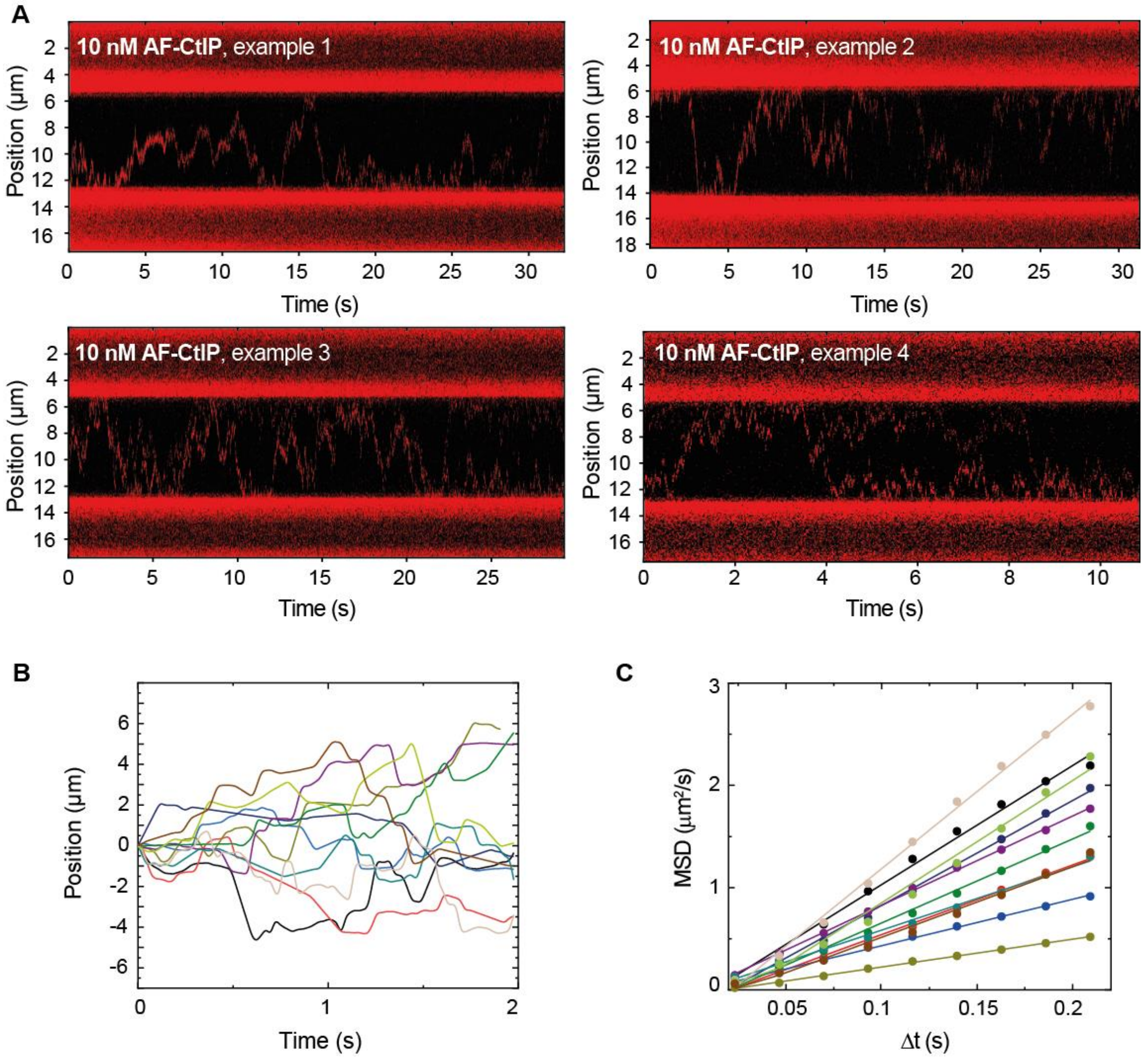

Supplementary Figure 7. Representative kymographs of individual trajectories of CtIP diffusion on DNA.

(A) Representative examples of kymographs showing trajectories of individual WT-CtIP proteins labelled with Alexa Fluor 635 (AF). (B) Position of CtIP over time determined from the analysis of CtIP kymographs (N =115). (C) Mean squared displacement (MSD) of CtIP for different time intervals (Δt). Straight lines indicate normal (i.e., random walk) diffusive behaviour.

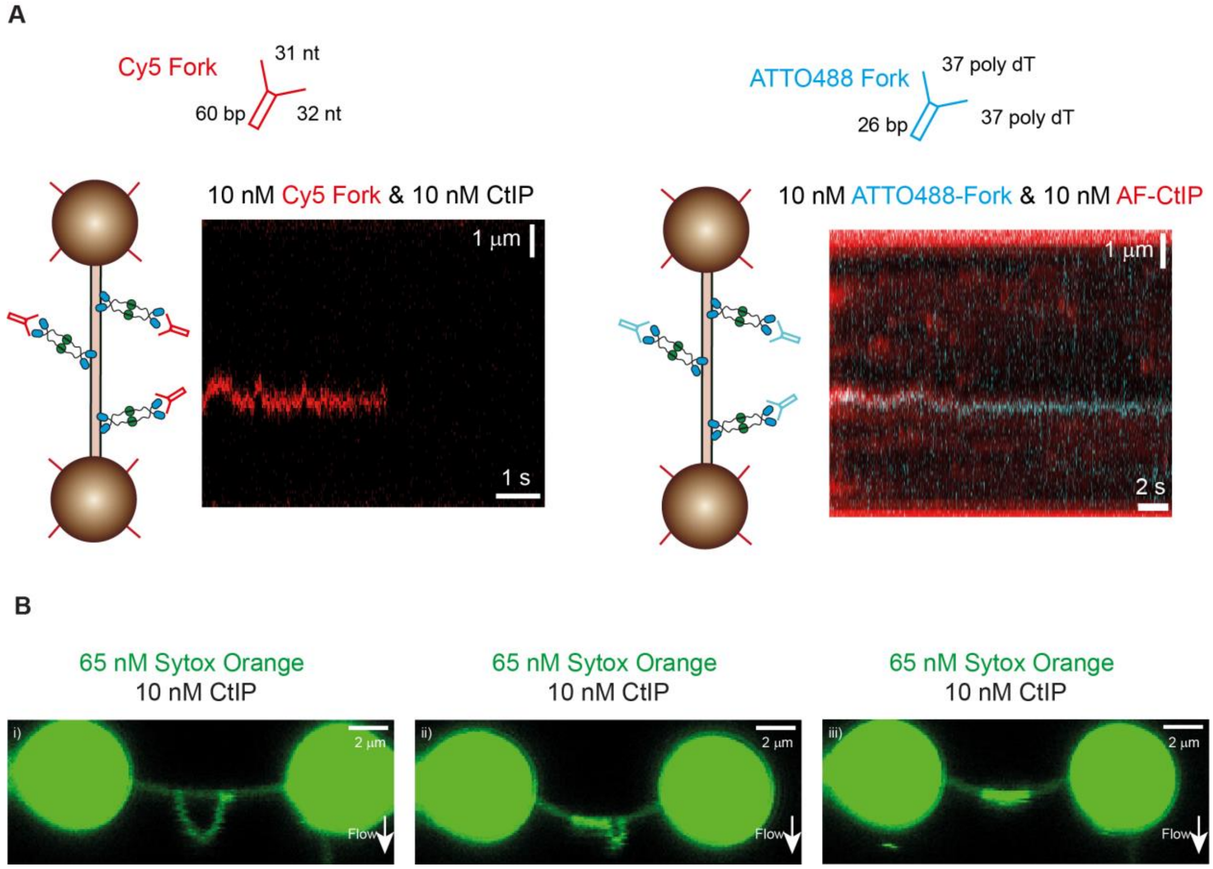

Supplementary Figure 8. DNA bridging in trans and in cis mediated by CtIP.

(**A, left**). Kymograph showing the DNA bridging of a Cy5 labelled fork DNA mediated by unlabelled CtIP. (**A, right**) Kymograph showing the DNA bridging of an ATTO488 labelled fork DNA mediated by labelled CtIP. (**B**) Control experiment using Sytox Orange to label the DNA and unlabelled CtIP. Three independent examples are shown. CtIP-mediated intramolecular bridging of the loop was observed upon application of a perpendicular flow to the tethered DNA.

Supplementary References

1. Aicart-Ramos C, Hormeno S, Wilkinson OJ, Dillingham MS, Moreno-Herrero F. Long DNA constructs to study helicases and nucleic acid translocases using optical tweezers. Methods Enzymol. 2022;673:311-58.
2. Fili N, Mashanov GI, Toseland CP, Batters C, Wallace MI, Yeeles JT, Dillingham MS, Webb MR, Molloy JE. Visualizing helicases unwinding DNA at the single molecule level. Nucleic Acids Res. 2010;38(13):4448-57.
3. Luzzietti N, Brutzer H, Klaue D, Schwarz FW, Staroske W, Clausing S, Seidel R. Efficient preparation of internally modified single-molecule constructs using nicking enzymes. Nucleic Acids Res. 2011;39(3):e15.
4. Too PH, Zhu Z, Chan SH, Xu SY. Engineering Nt.BtsCI and Nb.BtsCI nicking enzymes and applications in generating long overhangs. Nucleic Acids Res. 2010;38(4):1294-303.
5. Wilkinson OJ, Carrasco C, Aicart-Ramos C, Moreno-Herrero F, Dillingham MS. Bulk and single-molecule analysis of a bacterial DNA2-like helicase-nuclease reveals a single-stranded DNA looping motor. Nucleic Acids Res. 2020;48(14):7991-8005.
6. Crooks GE, Hon G, Chandonia JM, Brenner SE. WebLogo: a sequence logo generator. Genome Res. 2004;14(6):1188-90.
7. Drozdetskiy A, Cole C, Procter J, Barton GJ. JPred4: a protein secondary structure prediction server. Nucleic Acids Res. 2015;43(W1):W389-94.
8. Jumper J, Evans R, Pritzel A, Green T, Figurnov M, Ronneberger O, Tunyasuvunakool K, Bates R, Zidek A, Potapenko A, Bridgland A, Meyer C, Kohl SAA, Ballard AJ, Cowie A, Romera-Paredes B, Nikolov S, Jain R, Adler J, Back T, Petersen S, Reiman D, Clancy E, Zielinski M, Steinegger M, Pacholska M, Berghammer T, Bodenstein S, Silver D, Vinyals O, Senior AW, Kavukcuoglu K, Kohli P, Hassabis D. Highly accurate protein structure prediction with AlphaFold. Nature. 2021;596(7873):583-9.
